# Porous Aromatic Framework with Multifunctional Sites for Effective Recovery of Various Trace Iodine Species From Water

**DOI:** 10.1002/advs.202500993

**Published:** 2025-03-06

**Authors:** Yue Ma, Jinjiao Pan, Huazhen Rong, Yilei Zhang, Lu Liu, Yu Guo, Jiayi Ai, Yihui Yuan, Ning Wang

**Affiliations:** ^1^ State Key Laboratory of Marine Resource Utilization in South China Sea Hainan University Haikou 570228 P. R. China

**Keywords:** adsorption, iodine, iodine pollution, iodine resource, porous aromatic framework (PAF)

## Abstract

Recovery of environmental iodine is of great significance for both recycling iodine resources and addressing iodine pollution. However, iodine is highly sensitive to environmental factors and exists in various chemical species, which complicates the recovery of trace iodine in aqueous systems. Here a porous aromatic framework (iPAF‐TEPT) is presented with multifunctional adsorption sites for efficient recovery of various iodine species, including molecular iodine (I_2_), iodide (I^−^ and I_3_^−^). The material utilizes a synergistic strategy combining charge‐transfer interactions and Coulomb interactions to effectively adsorb different iodine species. Thanks to its high density of accessible ion exchange sites for I⁻ and I_3_⁻, and nitrogen‐rich sites for I_2_, iPAF‐TEPT demonstrates an unprecedented adsorption capacity for various iodine forms. Notably, iPAF‐TEPT achieves exceptional removal efficiency for trace iodine pollutants, even at concentrations as low as 100 ppb, making it the first promising single‐framework material for highly efficient treatment of aqueous iodine contamination.

## Graphical abstract

A PAF adsorbent with multifunctional sites framework is developed for the recovery of environment iodine. Benefiting from the high density and accessibility of the favorable Coulomb interaction sites for iodide (I_3_^−^ and I^−^) as well as the charge transfer sites for iodine molecules (I_2_), the PAF adsorbent realizes recording adsorption capacity for various iodine species.

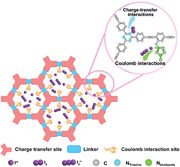

## 1 Introduction

Iodine is an important and scarce resource and is widely used for medical, industrial, agricultural, and food applications.^[^ ^1^ ^]^ With the rapid economic development, the demand for iodine resources is increasing.^[^ ^2^ ^]^ Seawater contains abundant iodine resources that can be extracted, yet the chemical technique for recovering iodine from seawater is still lacking. It is necessary and practical to explore new ways to obtain iodine resources from the environment. In medicine, chemical industry and other fields, iodine is used as a reaction intermediate that is not introduced into the target product but is discharged along with the wastewater, resulting in a negative impact on the ecological environment.^[^ ^3^ ^]^ Moreover, iodine pollution can result from the leakage of iodine‐containing ore and the use of iodine disinfectants, and long‐term intake of excessive iodine will have adverse effects on human health.^[^ ^4^ ^]^ In the nuclear industry, improper management and disposal of nuclear waste, and sudden nuclear accidents, can also release large amounts of radioactive iodine pollutants. The Fukushima nuclear disaster had led to the release of 1.6 × 10^17^ Bq L^−1^ radioactive species into the environment, and excessive amounts of radioactive iodine were detected in water purification plants, groundwater and rainwater, leading to a dramatic increase in the incidence of thyroid cancer and irreversible damage to the ecological environment.^[^ ^5^ ^]^ Recycling iodine from contaminated environments is a promising strategy for both removing iodine pollution and meeting iodine demand. Therefore, the recovery of environmental iodine is of great significance to the recycling of iodine resources and the treatment of environmental iodine pollution.

To efficiently recover the iodine pollutants from water, a series of porous materials, including metal organic frameworks (MOFs), covalent organic frameworks (COFs), hydrogen‐bonded organic frameworks (HOFs), and porous organic polymers (POPs), has been developed, which exhibit excellent performance for capturing high concentrations of iodine in water.^[^ ^4^, ^6^ ^]^ However, the concentration of iodine pollutants in real‐world scenarios, such as chemical industry wastewater, and contaminated seawater or groundwater, is generally less than 10 ppm, which makes most of the existing materials tricky to handle in these settings.^[^ ^4^, ^7^ ^]^ More importantly, iodine species are affected by environmental conditions such as pH and oxidation‐reduction potential in the water, making them diverse in the water environment. These species often exist as molecule (I_2_) or triiodide (I_3_^−^) in water, while the ionic form of iodine (I^−^) is the dominant species in weakly alkaline and reductive groundwater.^[^ ^3a,c^ ^]^ Furthermore, excess competing anions and extreme pH lead to troublesome operating conditions. Such complex iodine pollution systems make the recovery of low‐concentration environment iodine technically challenging, and simultaneous remediation of various trace iodine pollutants has not been demonstrated using a single porous framework material.

It is necessary to gain insight into the specific interactions between different iodine species and the functional groups of adsorbents at the molecular level, thereby further constructing a framework structure with a strong affinity and recognition for various iodine species and accomplishing effective capture. Charge‐transfer interactions between electron‐deficient iodine molecules and electron‐rich porous materials with optimized dense binding sites are conducive to iodine capture.^[^ ^8^ ^]^ Using this strategy, doped aromatic networks of heteroatoms and heterocycles, including triazines, pyridines, and amines, have shown clear benefits in improving I_2_ removal efficiency. Iodide can be trapped by Coulomb interactions, and cationic functional adsorbents can effectively enrich iodide.^[^ ^6^, ^9^ ^]^ Furthermore, iodine adsorption performance depends on the structural characteristics of the adsorbents, such as specific surface area and pore size.^[^ ^10^ ^]^ Therefore, the co‐assembling heterocyclic functional groups and cationic groups into a single framework with abundant porosity is a promising strategy for the effective recovery of various iodine species at low concentrations.

**Figure 1:**
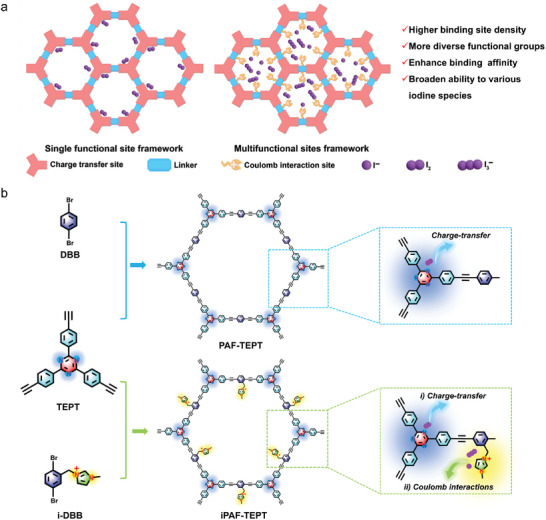
Design and synthesis of PAFs. a) Shortage and advantage of single functional adsorption site framework and multiple adsorption site framework. b) Synthesis of PAF‐TEPT and iPAF‐TEPT and the functional sites for binding different iodine species.

Porous aromatic frameworks (PAFs) represent extremely stable materials with an aromatic network structure connected by unique carbon‐carbon bonds, which allows them to be used in demanding environments, such as harsh pH conditions.^[^ ^11^ ^]^ The high specific surface area, abundant porosity, and flexible functionalization enable the assembly of PAFs with accessible affinity sites for different iodine species. Therefore, charge‐transfer and ion‐exchange functional sites with high affinity and density can be properly incorporated into the PAF structure, which satisfies the requirement for a single material to capture various iodine species at low concentrations. Based on these considerations, we herein designed a monomer with an imidazole cationic group and polymerized it with an electron‐rich triazine‐based monomer to obtain a PAF adsorbent (iPAF‐TEPT) for efficient iodine pollutants (I_2_, I^−^, and I_3_^−^) capture via the multifunctional‐site synergistic strategy by combining the charge‐transfer functional sites and Coulomb interaction site. The iPAF‐TEPT with multifunctional sites provides a high density of accessible favorable ion exchange sites for iodide (I_3_^−^ and I^−^), and imidazole‐N groups, as well as triazine‐N groups, can bind iodine molecules (I_2_) effectively and preferentially. Compared with PAF‐TEPT containing only triazine groups, the multifunctional‐site synergistic strategy endowed iPAF‐TEPT with a higher binding site density and more diverse functional groups, offering stronger affinity for multiple iodine species, which empowers iPAF‐TEPT with enhanced adsorption performance to aqueous I_2_, I^−^, and I_3_^−^ (**Figure** **1**a). Notably, iPAF‐TEPT can achieve a deep recovery effect for various trace iodine pollutants, even if the initial concentrations of the iodine pollutants are only 100 ppb, making it the first single framework material reported to recover various iodine pollutants at low concentrations. The developed iPAF‐TEPT, based on the multifunctional‐site synergistic strategy, exhibited excellent performance and applicability in diverse settings, indicating its potential as an adsorbent for various trace iodine pollutants under practical conditions.

## 2 Results

### 2.1 Synthesis and Structure Characterizations

In this study, multifunctional iPAF‐TEPT was synthesized using methylimidazolium‐modified dibromobenzene (i‐DBB) and 2,4,6‐tris (4‐ethynylphenyl) ‐1,3,5‐triazine (TEPT) as building units to embed imidazole cationic groups and triazine groups into the framework. The one‐pot synthetic route used in this study ensured an even distribution of the cationic groups within the framework, in contrast to the post‐modification route. PAF‐TEPT, which contains only triazine groups but lacks a cationic group, was synthesized using the building units dibromobenzene (DBB) and TEPT (Figure 1b; Figures S1 and S2, Supporting Information). The structural components of the as‐synthesized PAFs were investigated using Fourier transform infrared (FT‐IR) spectroscopy and ^13^C cross‐polarization magic angle spinning (CP‐MAS) NMR spectroscopy. The characteristic vibration of the terminal alkynyl C–H bond of TEPT disappeared in both iPAF‐TEPT and PAF‐TEPT. Additionally, the alkyne bonds exhibited a blue‐shift from 2 107 cm^−1^ in TEPT to 2 218 cm^−1^ in iPAF‐TEPT and 2 210 cm^−1^ PAF‐TEPT, indicating that the terminal alkynyl coupled with i‐DBB and DBB, respectively. Meanwhile, all vibrations of the imidazolium and triazine groups were retained, indicating the presence of potential iodine‐binding sites (Figure S3, Supporting Information). The characteristic carbon atoms in iPAF‐TEPT and PAF‐TEPT were assigned to their specific chemical shifts in the ^13^C CP‐MAS NMR spectra, which also demonstrated the successful fabrication of these materials (Figure S4, Supporting Information). Scanning electron microscopy (SEM) images revealed that the morphologies of iPAF‐TEPT and PAF‐TEPT exhibited spherical nanoparticles with sizes of hundreds of nanometers (Figure S5, Supporting Information). The porosities of iPAF‐TEPT and PAF‐TEPT were determined by nitrogen adsorption‐desorption isotherms collected at 77 K. According to the Brunauer‐Emmett‐Teller model, the BET surface area of iPAF‐TEPT (253.93 m^2^ g^−1^) was close to that of PAF‐TEPT (254.80 m^2^ g^−1^). Pore size distribution analysis shows that the pores of iPAF‐TEPT are predominantly concentrated ≈1.0 and 1.7 nm, while those of PAF‐TEPT are primarily concentrated ≈1.0, 1.7, and 3.1 nm. These results suggest that the introduction of imidazole cation sites has little to no impact on the porous properties of the framework (Figure S6, Supporting Information). The porous properties of the framework enhance iodine adsorption by facilitating the transport of aqueous iodine within the framework. Water contact angle measurements were conducted on iPAF‐TEPT and PAF‐TEPT to assess their hydrophilicity. The results revealed that the water contact angles of iPAF‐TEPT and PAF‐TEPT were 70° and 130°, respectively. These findings indicate that iPAF‐TEPT exhibits good water transport capabilities, making it well‐suited for use in aqueous environments (Figure S7, Supporting Information). Thermogravimetric analysis (TGA) was conducted to assess the thermal stabilities of iPAF‐TEPT and PAF‐TEPT. The results demonstrate that both materials exhibit excellent thermal stability (Figure S8, Supporting Information). Furthermore, iPAF‐TEPT remained intact after exposure to both acid and alkali treatments, as well as long‐term water immersion, highlighting its practical potential for iodine removal in natural water environments (Figure S9, Supporting Information).

### 2.2 Iodine Adsorption from Aqueous Environment

**Figure 2:**
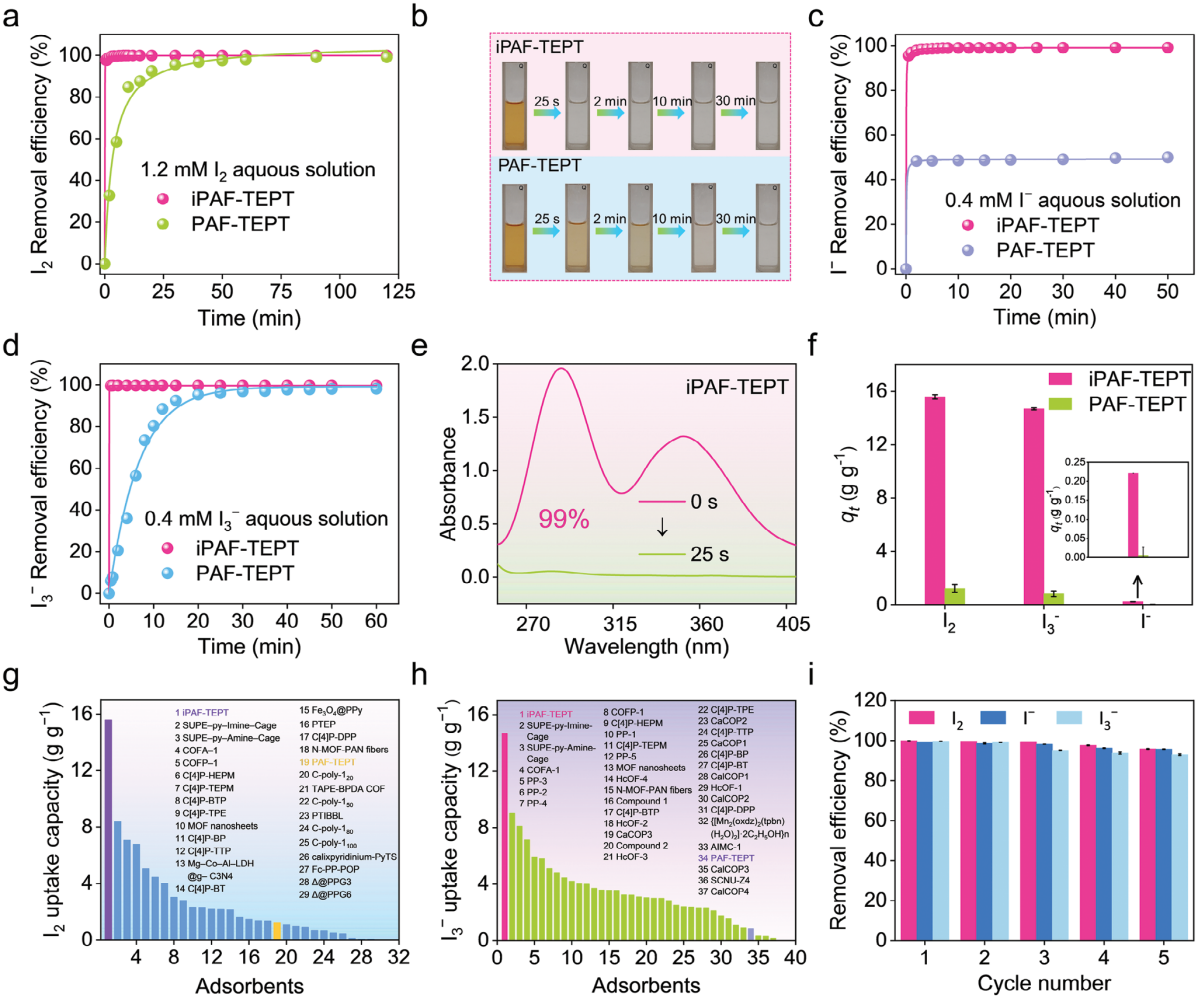
Iodine adsorption from aqueous solution. a) Time‐dependent adsorption models in 1.2 mM I_2_ aqueous solution. b) Color change of the I_2_ aqueous solution during the adsorption process. c) Time‐dependent adsorption models in 0.4 mm I^−^ aqueous solution. d) Time‐dependent adsorption models in 0.4 mm I_3_^−^ aqueous solution. e) Time‐dependent UV–vis spectra upon adding two adsorbents into 0.4 mm I_3_^−^ aqueous solution. f) The adsorption capacities of two adsorbents in three iodine species aqueous solutions. g) Comparison of I_2_ uptake capacities in aqueous solution with various reported adsorbents.^[^ ^3^, ^6^, ^12^ ^]^ h) Comparison of I_3_^−^ uptake capacities in aqueous solution with various reported adsorbents.^[^ ^3^, ^6^, ^12^, ^13^ ^]^ i) Reusability for different iodine species removal in aqueous solution.

Structurally, iPAF‐TEPT contains high‐density triazine and imidazole cationic groups. These N‐rich and cationic properties endow iPAF‐TEPT with potential efficient binding ability to diverse iodine species, including I_2_, I^−^, and I_3_^−^, while PAF‐TEPT, which contains only triazine groups, can only bind I_2_. To test this proposition, we performed adsorption experiments using I_2_, I^−^, and I_3_^−^ aqueous solution as separate source phase. Notably, iPAF‐TEPT demonstrated an impressive removal efficiency of ≈99% within 50 s in a 1.2 mm I_2_ aqueous solution, while PAF‐TEPT took 90 min to remove I_2_ and achieved a removal efficiency of 99% (**Figure** **2**a). Meanwhile, iPAF‐TEPT exhibited a noticeable and rapid color change after immersion in the I_2_ aqueous solution, with the color changing instantly from brown to colorless. In contrast, PAF‐TEPT caused a color change in the I_2_ aqueous solution over a period of at least 30 min (Figure 2b). According to the pseudo‐first‐order and pseudo‐second‐order kinetic model fitting results, the adsorption behaviors of iPAF‐TEPT and PAF‐TEPT were consistent with the pseudo‐second‐order kinetic model fitting, indicating that these two adsorbents absorbed aqueous I_2_ mainly via chemisorption (Figures S10 and S11, Supporting Information). Apart from I_2_, iodide (I^−^ and I_3_^−^) could also be the iodine pollutants found in wastewater and groundwater. Therefore, it is necessary to investigate the iodide removal performance of iPAF‐TEPT and PAF‐TEPT. We performed for I^−^ removal experiments with an initial I^−^ concentration of 0.4 mm and an adsorbent dosage of 1 g L^−1^. The result showed that over 99% of I^−^ was removed by iPAF‐TEPT, while PAF‐TEPT achieved a poor removal efficiency of 49%. This result indicates that the introduction of cationic sites into iPAF‐TEPT greatly improved its adsorption ability for I^−^ (Figure 2c; Figures S12 and S13, Supporting Information). In an aqueous medium, iodine tended to form poly‐iodides by the interaction between I^−^ and I_2_, among which triiodide (I_3_^−^) was one of the most predominant species. The adsorption performance of iPAF‐TPET and PAF‐TPET for aqueous I_3_^−^ at a dosage of 1 g L^−1^ has also been determined. iPAF‐TPET exhibited rapid removal efficiency of above 99% within 25 s in 0.4 mm I_3_^−^ aqueous solution, while PAF‐TEPT took as long as 40 min to achieve a removal efficiency of 98% (Figure 2d; Figures S14 and S15, Supporting Information). The intensity of the characteristic peak of I_3_^−^ in the ultraviolet‐visible (UV–vis) spectra of the I_3_^−^ aqueous solution decreased sharply with the extension of the adsorption time during the adsorption of I_3_^−^ by iPAF‐TEPT, proving its high adsorption efficiency (Figure 2e).

To evaluate the optimum adsorption capacities of iPAF‐TEPT and PAF‐TEPT for three iodine species, the equilibrium adsorption capacities of iPAF‐TEPT and PAF‐TEPT at a dose ratio of 0.01 g L^−1^ for aqueous I_2_, I^−^, and I_3_^−^ were determined (Figure 2f). The results of adsorption experiments showed that the equilibrium adsorption capacity of iPAF‐TEPT was 15.59, 14.68, and 0.22 g g^−1^ for I_2_, I_3_^−^, and I^−^, respectively, which was found to be much higher than that of PAF‐TEPT for I_2_ (1.22 g g^−1^), I_3_^−^ (0.83 g g^−1^), and I^−^ (0.02 g g^−1^). Most impressively, the I_2_ and I_3_^−^ adsorption capacities from aqueous solution surpassed those of most other adsorbents reported to date (Figure 2g,h; Tables S1 and S2, Supporting Information). These results clearly reflect the advantages of framework materials with multiple functional sites over those with a single functional site for the efficient adsorption of diverse iodine species. The reusability of the adsorbents is crucial for sustainable and cost‐effective practical applications. It is worth mentioning that the iodine species adsorbed on iPAF‐TEPT were easily desorbed by ethanol. As shown in Figure 2i, the removal efficiencies of iPAF‐TEPT for I_2_, I^−^, and I_3_^−^ showed almost no significant decrease after four times of reuse.

### 2.3 Iodine Adsorption from Complex Water Environments

**Figure 3:**
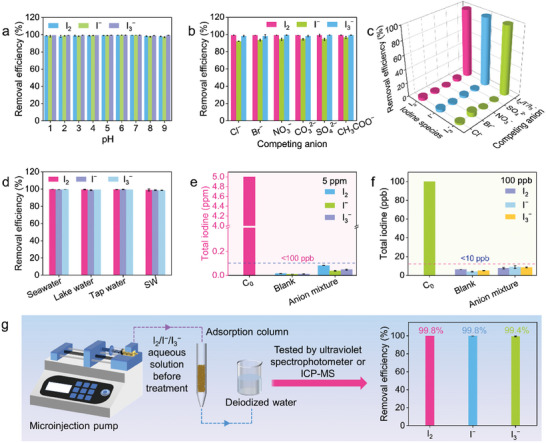
Iodine adsorption from complex water environments by iPAF‐TEPT. a) The effect of pH on the adsorption of three iodine species. b) Influence of individual 1 000 equivalent competing anions on the adsorption of different iodine species. c) Influence of 100 equivalent co‐existing competing anions on the adsorption of different iodine species. d) Removal efficiency to different iodine species from four iodine‐containing water sources. e) The adsorption of different iodine species from aqueous solution and aqueous solution containing 10 equivalent anion mixtures with an initial iodine species concentration of 5 ppm. f) The adsorption of different iodine species from aqueous solution and aqueous solution containing 10 equivalent anion mixtures with an initial iodine species concentration of 100 ppb. g) Column breakthrough adsorption ability to different iodine species by column breakthrough adsorption scheme.

Considering the application challenges of aqueous iodine adsorption in real‐world scenarios, such as extreme pH and abundant competing anions, we further explored the ability of iPAF‐TEPT to capture diverse iodine pollutants from harsh environments. First, we evaluated the effect of pH on the iodine removal efficiency of iPAF‐TEPT in 1.2 mm I_2_, 0.4 mm I^−^, and 0.4 mm I_3_^−^ aqueous solution. As shown in **Figure** **3**a, the iodine removal efficiency of iPAF‐TEPT was higher than 98% for I_2_, and higher than 99% for I_3_^−^ from pH 1 to pH 9. Satisfyingly, the removal efficiency of iPAF‐TEPT for I^−^ was ≈99% at pH from pH 1 to pH 7, and higher than 97% at pH 8 and 9. As a result, iPAF‐TEPT with multiple functional sites still exhibited a strong affinity for three iodine species over a wide pH range, even in highly acidic conditions with pH from pH 1 to pH 3. Competitive adsorption experiments were carried out in the presence of 1 000 equivalents of interfering anions, including Cl^−^, Br^−^, NO_3_^−^, CO_3_^2−^, SO_4_^2−^, and CH_3_COO^−^, at equal‐molar concentration to evaluate the selectivity of iPAF‐TEPT toward iodine species over other interfering anions. The I_2_ removal efficiency of iPAF‐TEPT remained essentially unchanged at ≈99%. Furthermore, the removal efficiencies for I^−^ and I_3_^−^ were only slightly affected by interfering anion and removal efficiency of 92% and 98%, respectively (Figure 3b). In comparison, PAF‐TEPT exhibited poor I_2_ and I_3_^−^ removal efficiencies of ≈50% and 40%, respectively, and I^−^ removal efficiency of less than 23% under the same condition (Figure S16, Supporting Information). In addition, iPAF‐TEPT exhibited high removal efficiencies of 98%, 98%, and 99% for I_2_, I^−^, and I_3_^−^, respectively, in the presence of 100‐fold competing anions, including Cl^−^, Br^−^, NO_3_^−^, and SO_4_^2−^. It is worth noting that the adsorption of interfering anions by iPAF‐TEPT was negligible, even when the molar ratio of the competing anions to iodine species reached 100 (Figure 3c). These results demonstrate the high adsorption selectivity of iPAF‐TEPT for diverse iodine species.

Motivated by the highly efficient and selective removal of diverse iodine species by iPAF‐TEPT, we determined the adsorption capacities of different iodine species in simulated iodine‐polluted water environments, including seawater, lake water, tap water, and simulated groundwater (SW). In all the water systems used, iPAF‐TEPT showed excellent removal efficiencies higher than 99%, 97%, and 98% for I_2_, I^−^, and I_3_^−^, respectively (Figure 3d). These results indicate that iPAF‐TEPT is an impressive adsorption material for capturing iodine species from natural water sources, making it a promising solution for the recovery of environmental iodine.

In practical application scenarios, iodine pollutant concentrations are generally low. We then performed adsorption experiments to explore the application potential of iPAF‐TPET from aqueous solutions containing trace amounts of I_2_, I^−^, and I_3_^−^. As shown in Figure 3e, after being treated with iPAF‐TEPT, the residual I_2_, I^−^, and I_3_^−^ concentrations were measured at 15.4, 9.8, and 11.4 ppb, respectively, in the iodine solution with an initial concentration of 5 ppm. Excitingly, the residual iodine species in the complex aqueous solutions were measured as low as a ppb‐level of 83 ppb for I_2_, 37 ppb for I^−^, and 48 ppb for I_3_^−^ in the presence of 10 equivalent co‐existing competing anions (Cl^−^, Br^−^, NO_3_^−^, SO_4_^2−^). Notably, for the ultra‐low initial iodine species concentration of 100 ppb, the residual concentrations of I_2_, I^−^, and I_3_^−^ were only 6.2 ppb, 4.1 ppb, and 5.1 ppb, respectively. Moreover, the residual concentrations of I_2_, I^−^, and I_3_^−^ were all less than 10 ppb, even in the presence of 10 equivalent co‐existing competing anions (Cl^−^, Br^−^, NO_3_^−^, SO_4_^2−^) (Figure 3f). To the best of our knowledge, these results reflect the unprecedented advantage of selective capture of aqueous iodine pollutants by multiple functional site materials and iPAF‐TEPT represents the first single framework to capture various iodine species at low concentrations. To broaden the potential application of the adsorbent in nuclear wastewater treatment, we focused on conditions that closely mimic the acidic environment typically encountered during the acid leaching step of spent fuel reprocessing. Given that nuclear wastewater generated in this process typically exhibits an acidic pH, it is crucial to assess the adsorbent's performance under such conditions. To this end, we conducted adsorption experiments for trace iodine (5 ppm and 100 ppb, including I_2_, I^−^, and I_3_^−^) across a pH range of 1 to 6. The results indicated that, at an iodine concentration of 5 ppm, the removal efficiency was reduced to 110 ppb, while at 100 ppb, the iodine removal dropped further to below 20 ppb (Figure S17, Supporting Information). These findings suggest that, although the removal efficiency decreased under acidic conditions, the adsorbent still demonstrated effective performance in removing trace iodine. This reinforces its potential applicability in nuclear wastewater treatment, even under the acidic pH conditions typically encountered during spent fuel reprocessing. The reusability of iPAF‐TEPT for treating trace iodine pollutants was evaluated to assess its practical application potential. The results showed that removal efficiencies of iPAF‐TEPT for I_2_, I^−^, and I_3_^−^, with an initial concentration of 5 ppm, remained above 99% after four cycles of reuse. At an I_2_, I^−^, and I_3_^−^ concentration of 100 ppb, there was almost no significant decline in removal efficiencies, demonstrating the excellent reproducibility of iPAF‐TEPT (Figure S18, Supporting Information). To further assess the performance of the adsorbent in practical applications, a series of cyclic tests were conducted using I_2_, I^−^, and I_3_^−^ solutions at initial concentrations of 5 ppm and 100 ppb, with the addition of 10 equivalent concentrations of competing anions (Cl^−^, Br^−^, NO_3_^−^, SO_4_^2−^). The results demonstrated that, for the three iodine solutions at an initial concentration of 5 ppm, the removal efficiency remained above 95% after five cycles. Furthermore, at a concentration of 100 ppb, the removal efficiency remained above 80% after five cycles (Figure S19, Supporting Information). These findings indicate that iPAF‐TEPT exhibits excellent reusability for trace iodine removal in the presence of competing anions.

Inspired by the efficient and selective capture of various iodine species from water, we speculated that iPAF‐TEPT could be a potential adsorbent for the dynamic capture of iodine pollutants. The dynamic flow‐through experiments were carried out in the presence of 100 equivalent co‐existing competing anions (Cl^−^, Br^−^, NO_3_^−^, SO_4_^2−^). First, a column with a 4 mm^2^ cross‐sectional area was filled with iPAF‐TEPT to a height of 2 cm. Thereafter, 1.2 mm I_2_, 0.4 mm I^−^, or 0.4 mm I_3_^−^ aqueous solution was passed through the column at a flow rate of 0.3 mL min^−1^, respectively. Subsequently, the brown I_2_ aqueous solution or the yellow I_3_^−^ aqueous solution turned completely colorless after passing through the column, and the removal efficiency of iPAF‐TEPT was calculated as 99.8% for I_2_ or 99.4% for I_3_^−^ using UV–vis spectra. For I^−^ polluted water, iPAF‐TEPT also exhibited excellent removal efficiency of 99.8%, as detected by ICP‐MS (Figure 3g). We further conducted dynamic breakthrough experiments to assess the practicality of iPAF‐TEPT for removing trace iodine. After the breakthrough in an iodine solution with an initial concentration of 5 ppm, and in the presence of 10 equivalents of competing anions (Cl^−^, Br^−^, NO_3_^−^, SO_4_^2−^), the residual concentrations of I_2_, I^−^, and I_3_^−^ were found to be below 100 ppb. For an ultra‐low initial iodine concentration of 100 ppb, the residual concentrations of I_2_, I^−^, and I_3_^−^ were all under 15 ppb, demonstrating the promising potential of iPAF‐TEPT for treating iodine‐polluted water (Figure S20, Supporting Information). The excellent dynamic iodine capture performance indicates the high potential of iPAF‐TEPT for the treatment of aqueous iodine pollutants in industrial applications.

### 2.4 Characterization and Interaction Mechanism Studies

**Figure 4:**
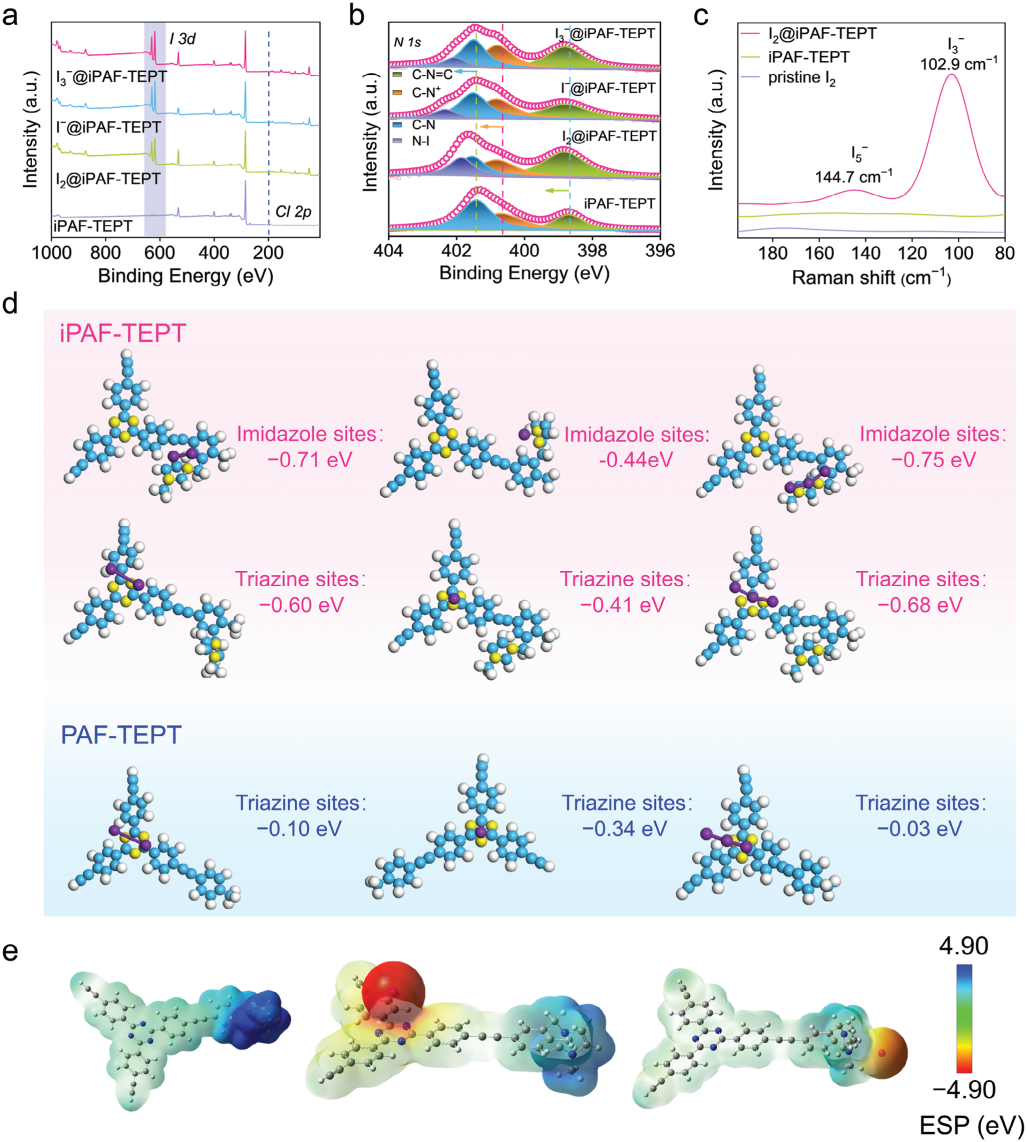
Characterization and interaction mechanism of iPAF‐TEPT with three iodine species. a) Full XPS spectrum of iPAF‐TEPT before and after iodine adsorption. b) High‐resolution XPS for N *1s* of iPAF‐TEPT before and after iodine adsorption. c) Roman spectra of iodine‐loaded iPAF‐TEPT. d) Structural models fragment of three iodine species interacting with different N sites in iPAF‐TEPT and PAF‐TEPT and the corresponding calculated adsorption energies. e) The electrostatic potential distributions for iPAF‐TEPT, the triazine site of iPAF‐TEPT after iodide uptake, and imidazole of iPAF‐TEPT after iodide uptake.

The experimental results showed that iPAF‐TEPT exhibited excellent removal performances for I_2_, I^−^, and I_3_^−^. To uncover the adsorption mechanism of diverse iodine species, we performed detailed characterization studies on iPAF‐TEPT and iodine‐loaded iPAF‐TEPT. Transmission electron microscopy (TEM) and the corresponding elemental mapping images displayed that iodine was distributed evenly in iPAF‐TEPT after the adsorption process (Figure S21, Supporting Information). New I *3d* peaks were observed in the XPS spectra of iPAF‐TEPT after the adsorption of I_2_, I^−^, and I_3_^−^. It is worth noting that the peak of Cl *2p* was weakened significantly due to the ion exchange of the Cl counter anion by I^−^ or I_3_^−^. In the case of the I_2_, the charge‐transfer complexes that formed between I_2_ and the N‐sites caused ion exchange with free Cl^−^ (**Figure** **4**a). By comparing the N *1s* high‐resolution XPS spectra of iPAF‐TEPT before and after adsorption, the adsorption of I_2_ on iPAF‐TEPT caused the shift of the peaks for C = N and C–N from 398.7 eV to 398.8 eV and 401.4 eV to 401.5 eV, respectively, revealing the charge‐transfer complexes formation between the captured I_2_ and N‐sites of triazine‐N sites and imidazole‐N sites. Meanwhile, the peak assigned to the imidazole‐N^+^ sites (N^+^–C) shifted from 400.7 to 400.8 eV, indicating that they were involved in forming new complexes with I_2_ via Coulomb interaction. Additionally, a new N–I related peak appeared at 401.8 eV, indicating the interaction between I_2_ and the triazine‐N sites and imidazole‐N sites (including imidazole‐N^+^ sites) of iPAF‐TEPT. In the case of I^−^ and I_3_^−^, similar peak shifts and weaker signals were observed, suggesting that the formation of new complexes also probably occurred between I^−^ or I_3_^−^ with triazine‐N sites and imidazole‐N sites (including imidazole‐N^+^ sites) of iPAF‐TEPT (Figure 4b). Raman spectroscopy showed that two bands at 102.9 cm^−1^ and 144.7 cm^−1^ were ascribed to the stretching vibrations of I_3_^−^ and I_5_^−^, respectively, further suggesting that the charge‐transfer complexes formed between the captured I_2_ and iPAF‐TEPT (Figure 4c). After the adsorption of I_2_, the enhancement of the paramagnetic signal in the EPR spectra could also indicate the charge‐transfer interaction between the electric‐rich fragments of iPAF‐TEPT and the captured I_2_ (Figure S22, Supporting Information). These results established the mechanism of I_2_, I^−^, and I_3_^−^ adsorption by iPAF‐TEPT based on the synergistic interaction of multifunctional sites, including the charge transfer interaction between iodine species and electron‐rich heteroatomic groups, as well as the Coulomb interaction with cation sites of imidazole‐N^+^ to achieve enhanced affinity for various iodine species.

Additionally, DFT calculations further verified the interactions between iPAF‐TEPT and iodine species. The structural fragment of PAF‐TEPT or iPAF‐TEPT was used as a model to calculate the binding energy between the materials and I_2_, I^−^, and I_3_^−^. The calculation results showed that the triazine group of iPAF‐TEPT had a higher adsorption potential for I_2_, I^−^, and I_3_^−^, with binding energies of −0.60 eV, −0.41 eV, and −0.68 eV, respectively, compared to the binding energies of −0.10 eV, −0.34 eV, and −0.03 eV between the triazine group in PAF‐TEPT with I_2_, I^−^, and I_3_^−^, respectively. Furthermore, the binding energies between I_2_, I^−^, and I_3_^−^ with imidazole cationic sites in iPAF‐TEPT were calculated to be −0.71 eV, −0.44 eV, and −0.75 eV, respectively (Figure 4d). These results suggest that cation functionalization sites increase the strength and number of binding sites for capturing iodine species through Coulomb interactions, resulting in a stronger affinity of iPAF‐TEPT for the iodine species. The electrostatic potential (ESP) of iPAF‐TEPT before and after the capture of I^−^ was analyzed. The results showed that the imidazole cationic groups of iPAF‐TEPT bind I^−^ more readily, which further explained the superior adsorption of I^−^ at imidazole cationic sites compared with other sites (Figure 4e).

The experimental and theoretical results illustrate that the developed iPAF‐TEPT with multifunctional sites is an excellent adsorbent for recovering multiple trace iodine pollutants under diverse complex conditions. Compared to PAF‐TEPT with a single functional site, iPAF‐TEPT with multifunctional sites composed of triazine groups and imidazole cation groups can enhance the affinity of I_2_, I^−^, and I_3_^−^, which suggests that the multifunctional‐site synergistic strategy, combining charge‐transfer interaction and Coulomb interaction, is more favorable for the adsorption of I_2_, I^−^, and I_3_^−^. Furthermore, iPAF‐TEPT can rapidly capture multiple iodine pollutants in the presence of other competing anions and even in natural water sources. This is mainly due to the high binding energies at the triazine and imidazole sites, as well as the high porosity that allows mass transport.

## 3 Conclusion

In summary, we report a PAF material iPAF‐TPET with multifunctional sites for efficient iodine pollutants (I_2_, I^−^, and I_3_^−^) capture via the multifunctional‐site synergistic strategy by combining the triazine and imidazole groups. Compared with PAF‐TEPT containing only triazine groups, the “multifunctional‐site synergistic” strategy, combined with charge transfer interaction and Coulomb interaction, endowed iPAF‐TEPT with higher binding site density and more diverse functional groups, offering stronger affinity to multiple iodine species, thereby empowering iPAF‐TEPT with a record‐breaking adsorption capacity from I_2_ aqueous solution (15.59 g g^−1^) and the reported highest adsorption capacity for aqueous I_3_^−^ (14.68 g g^−1^). Impressively, the removal efficiency of iPAF‐TEPT in an I^−^ aqueous solution exceeds 95% within just 30 s. More importantly, iPAF‐TEPT exhibited a deep removal ability for various trace iodine pollutants at the extremely low concentration of 100 ppb, representing the first single‐framework material reported to selectively capture various iodine pollutants at low concentrations. Characterization and theoretical calculations revealed that the rapid and selective adsorption of various iodine species by iPAF‐TEPT was intrinsically related to the high binding energies between the multifunctional sites and the iodine species. The developed iPAF‐TEPT, based on multifunctional‐site synergistic strategy, exhibits excellent performance and applicability in diverse settings, indicating its potential as an adsorbent for various trace iodine pollutants under practical conditions.

## Conflict of Interest

The authors declare no conflict of interest.

## Supporting information



Supporting Information

## Data Availability

The data that support the findings of this study are available from the corresponding author upon reasonable request.
