# Supplementary material for: Porous Aromatic Framework with Multifunctional Sites for Effective Recovery of Various Trace Iodine Species From Water
Source: Adv Sci (Weinh). 2025 Mar 6;12(17):2500993. doi: 10.1002/advs.202500993 (PMC12061248; doi:10.1002/advs.202500993)
Supplement: Supplementary file 1 — Supporting Information [file ADVS-12-2500993-s001.docx]

Supporting Information

Porous Aromatic Framework with Multifunctional Sites for Effective Recovery of Various Trace Iodine Species from Water

*Yue Ma^1,2^, Jinjiao Pan^1,2^, Huazhen Rong^1^, Yilei Zhang^1^, Lu Liu^1^, Yu Guo^1^, Jiayi Ai^1^, Yihui Yuan^1^* & Ning Wang^1^**

^1^State Key Laboratory of Marine Resource Utilization in South China Sea

Hainan University

Haikou 570228, P. R. China.

^2^These authors contributed equally: Yue Ma, Jinjiao Pan.

*Corresponding authors

Email: wangn02@foxmail.com; yuanyh@hainanu.edu.cn

**Materials**

All reactants and solvents used in this study were purchased from commercial suppliers and were not purified further. An aqueous solution of the iodine species was prepared using deionized water. Seawater was collected from the west coast of Haikou, Hainan Province. Water was collected from Dongpo Lake, Hainan University. Tap water was collected from the laboratory taps. The natural water sources must be filtered using a 0.22 μm filter membrane before use.

**Characterization**

^1^H NMR spectra were recorded using a nuclear magnetic resonance apparatus. ^13^C cross-polarization magic-angle spinning (CP/MAS NMR) spectroscopy was performed using a Bruker BioSpin GmbH instrument. Fourier-transform infrared (FT-IR) spectra were recorded using a PerkinElmer Fourier-transform infrared spectrometer. A Verios G4 UC field-emission scanning electron microscope (SEM) was used to observe the surface morphologies of the two prepared adsorbents. N_2_ adsorption and desorption at 77 K were determined using an American Mike ASAP2460 automatic. The two adsorbents were degassed at 120 °C under vacuum for 12 h before the N_2_ adsorption and desorption analyses. The UV-Vis spectra and absorbance were recorded using a UV-1600 spectrophotometer. Inductively coupled plasma (ICP) measurements were performed using a Thermo Scientific ICAP RQ ultrasensitive inductively coupled plasma emission mass spectrometer. The anion content was determined using ion chromatography (ICS-1500). Elemental analysis of iPAF-TEPT after iodine adsorption was performed using high-resolution transmission electron microscopy (HRTEM, JEM-2100). Raman spectroscopy was conducted using a Renishaw PLC laser Raman spectrometer. X-ray photoelectron spectroscopy (XPS) was performed using a Thermo SCIENTIFIC Nexsa spectrometer. Electron paramagnetic resonance (EPR) spectroscopy was performed on a Bruker A300-10/12 spectrometer.

**Synthesis of i-DBB.**

The i-DBB was synthesized referring to previously reported methods. ^[1]^ 2, 5-dibromotoluene (3.0 g, 12 mmol) (Ark Pharm), N-bromosuccinimide (2.7785 g, 15.62 mmol) (3A Chemicals) and benzoyl peroxide (0.0784 g, 0.24 mmol) (3A Chemicals) in 250 mL 2-necked round bottom flask equipped with magnetic stirring rod, 100 mL CCl_4_ was added, then stirred in a nitrogen atmosphere to heat reflux for about 18 h. The mixture was cooled to room temperature and filtered to remove the solids to produce a filtrate, which was distilled under vacuum to produce a yellow, oily substance. The crude product was purified by column chromatography to obtain compound 1. Compound 1 (0.5 g, 1.52 mmol), N-methylimidazole (0.188 g, 2.28 mmol of organic matter), placed in a 100 mL 2-necked round-bottomed flask equipped with a magnetic stirring rod, then added to dissolve in 30 mL CH_2_Cl_2_. The mixture was stirred in a nitrogen atmosphere and heated for reflux for about 21 h. Vacuum distillation of the solution into a small amount of remaining white oily liquid, adding ethyl acetate, precipitating salt, filtration and vacuum drying for 3 h to obtain i-DBB (white powder). ^1^H NMR (499.8 MHz, CDCl_3_): δ 4.13 (3H, CH_3_), 5.80 (2H, CH_2_Br), 7.47 (1H, Ar-H), 7.52 (1H, Ar-H), 7.54 (1H, Ar-H), 7.97 (2H, Imidazol-H), 10.96 (1H, Imidazol-H).

**
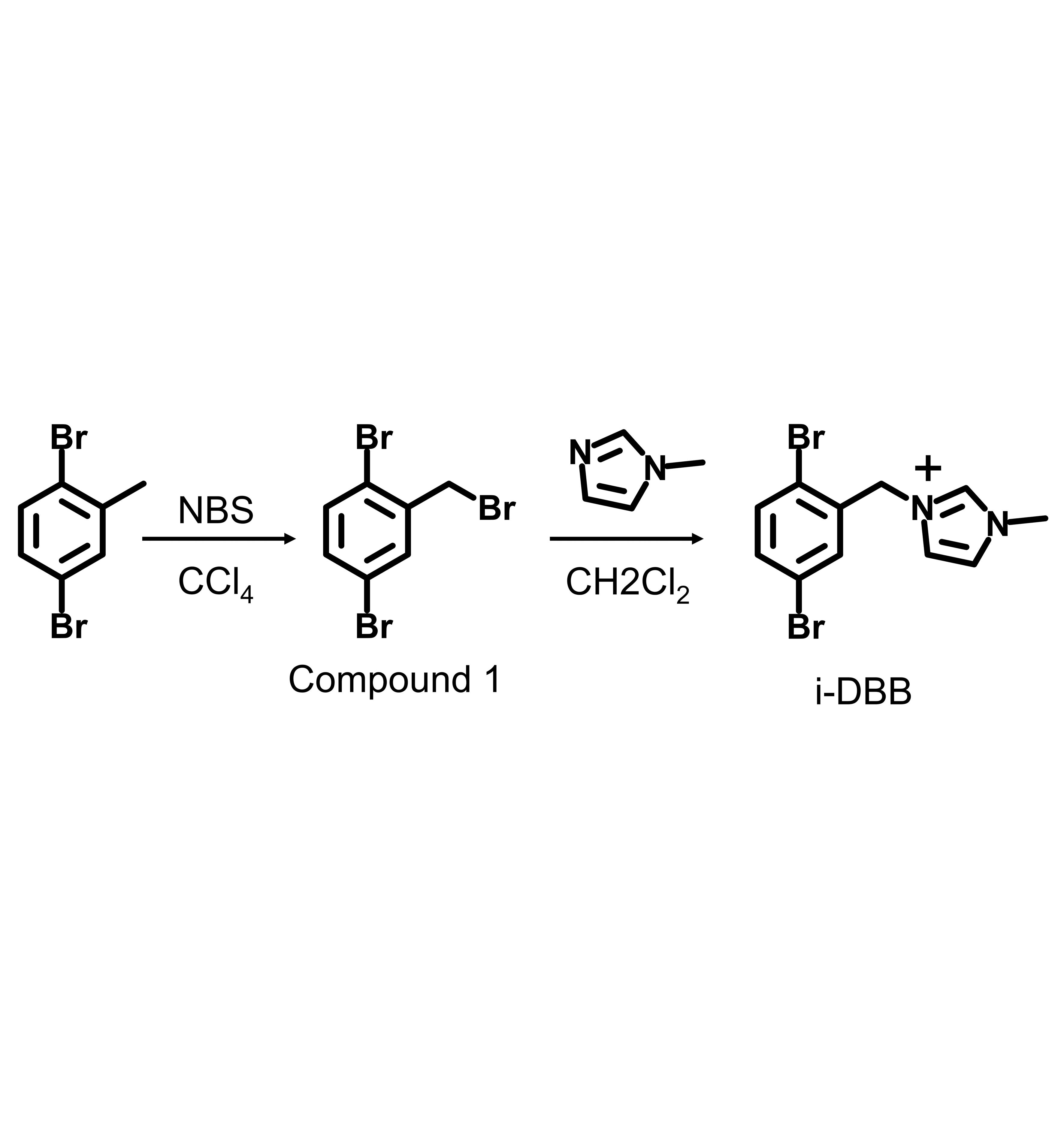
**

**Figure S1.** Schematic routes to the synthesis of i-DBB.


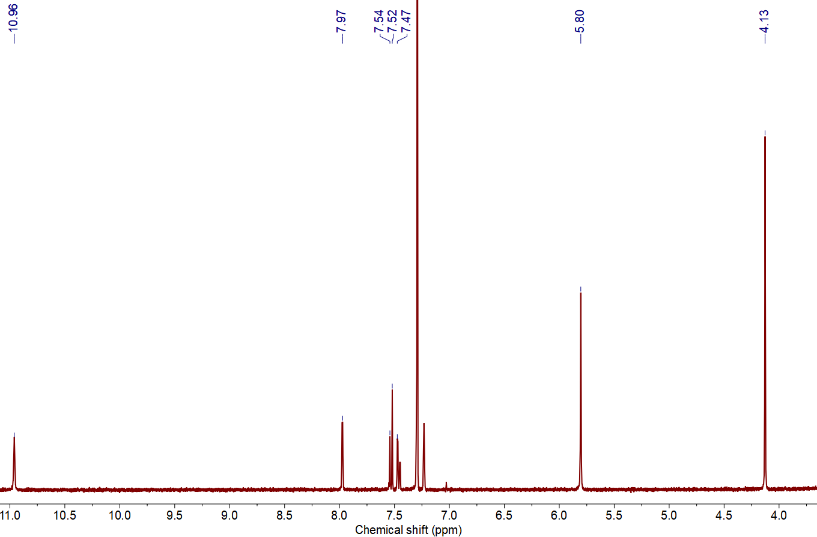


**Figure S2.** ^1^H NMR spectrum of i-DBB.

**Synthesis of iPAF-TEPT and PAF-TEPT**

Under a nitrogen atmosphere, weigh 0.023 g of CuI (0.12 mmol), 0.138 g of tetrakis(triphenylphosphine)palladium (0.12 mmol), 0.763 g of TEPT (2 mmol), and 0.99 g of i-DBB (3 mmol) or 0.708 g of DBB (3 mmol), and transfer the mixture into a 250 mL round-bottom flask. Add 100 mL of a 1:1 mixture of DMF and triethylamine as the solvent. Stir the reaction mixture at 120 °C for 48 hours, then allow it to cool to room temperature. After cooling, filter the solution and wash the resulting product sequentially three times with 6 M HCl, methanol, CH_2_Cl_2_, and ethanol. Finally, filter the product and dry it under vacuum in an 80 °C oven overnight to obtain a brown powder. The yields of iPAF-TEPT and PAF-TEPT are 87% and 90%, respectively.

**Iodine and Iodide adsorption from aqueous solution**

The adsorbent was immersed in an aqueous iodine solution (I_2_: 1.2 mM; I^−^: 0.4 mM, I_3_^−^: 0.4 mM), and stirred at at a rate of 1000 rpm room temperature, sampled at different times, and filtered using a 0.2 μm polyether sulfone (PES) membrane. The residual iodine concentration in the filtrate was measured using UV-Vis spectroscopy or ICP-MS. All adsorption experiments in this study were conducted under dark conditions to prevent the effects of light exposure. The experiments were carried out using either tinfoil wraps or amber-colored glassware, which effectively block light, particularly ultraviolet radiation that could decompose iodine or interfere with the adsorption process. The iodine-removal rate was calculated as follows:

$$\frac{C_{0}-C_{t}}{C_{0}}\times100\% ,$$

where $C_{0}$ (mM) and $C_{t}$ (mM) are the iodine concentrations before and after adsorption, respectively.

The capture capacity of iodine was calculated as

$q_{t}$=$\frac{{(C}_{0}-C_{t}) M_{w} V}{m}$ ,

where $q_{t}$ (g g^−1^) represents the amount of iodine adsorbed per gram of adsorbent at time$t$ (min). $C_{0}$(mM) and $C_{t}$ (mM) are the initial and residual concentrations of iodine in the stock solution and filtrate, respectively; $V$ (L) is the volume of the iodine solution, and $m$ (g) is the mass of the adsorbent used in the study. $M_{w}$ (g mol^−1^) is the molar mass of iodine.

The kinetic models of the adsorption experiments were the Ho and McKay pseudo-second-order kinetics, expressed as

$\frac{t}{q_{t}}=\frac{t}{q_{e}}+\frac{1}{k_{2}q_{e}^{2}}$,

where $q_{e}$ (g g^−1^) represents the amount of iodine adsorbed at equilibrium, $q_{t}$ (g g^−1^) represents the amount of iodine adsorbed at $t$ (min), $k_{2}$(g g^−1^min^−1^) is the pseudo-second-order kinetic constant, which was calculated by the intercept and slope of the corresponding curve with $\frac{t}{q_{t}}$ as the y axis and $t$ as the x axis.

**Iodine adsorption from I_2_, I^−^ or I_3_^−^ aqueous solutions supplemented with different competing anions**

iPAF-TEPT was added to 1000-fold excess of single competing anions (equal-molar Cl^−^, Br^−^, NO_3_^−^, CO_3_^2−^, SO_4_^2−^ or CH_3_COO^−^) and 100-fold excess of mixture co-existing competing anions (equal-molar Cl^−^, Br^−^, NO_3_^−^, and SO_4_^2−^) supplemented with 1.2 mM I_2_, 0.4 mM I^−^ or 0.4 mM I_3_^−^ respectively, stirred at a rate of 1000 rpm at room temperature, sampled, and filtered using a 0.2 μm PES membrane at different times. The residual iodine concentration in the filtrate was measured by ICP-MS. The concentrations of competitive anions in the filtrate were determined using ion chromatography.

**Iodine adsorption from I_2_, I^−^ or I_3_^−^ aqueous solutions at different pH**

The pH was adjusted using hydrochloric acid and sodium hydroxide aqueous solutions in 1.2 mM I_2_, 0.4 mM I^−^ or 0.4 mM I_3_^−^ aqueous solutions. iPAF-TEPT was added to I_2_, I^−^ or I_3_^−^ aqueous solution with pH = 1−9, stirred at a rate of 1000 rpm at room temperature, sampled, and filtered using a 0.2 μm PES membrane at different times. The residual iodine concentration in the filtrate was measured by ICP-MS.

**Iodine adsorption from three natural water and simulated groundwater (SW)**

SW is made according to the following distribution^[2]^: silicic acid (H_2_SiO_3_∙nH_2_O) 15.3 mg L^−1^, potassium chloride (KCl) 8.2 mg L^−1^, magnesium carbonate (MgCO_3_) 13.0 mg L^−1^, sodium chloride (NaCl) 15.0 mg L^−1^, calcium sulfate (CaSO_4_) 67.0 mg L^−1^, calcium carbonate (CaCO_3_) 150.0 mg L^−1^. iPAF-TEPT was added to the filtered seawater, lake water, tap water and SW supplemented with 1.2 mM I_2_, 0.4 mM I^−^ or 0.4 mM I_3_^−^ aqueous solutions, stirred at a rate of 1000 rpm at room temperature, sampled, and filtered using a 0.2 μm PES membrane at different times. The residual iodine concentration in the filtrate was measured using ICP-MS.

**Dynamic flow-through experiment**

The adsorbent was placed in the filling column at a height of 2 cm. The saturated iodine solution (1.2 mM I_2_, 0.4 mM I^−^, or 0.4 mM I_3_^−^) supplemented with a 100-fold excess of multiple co-existing competing anions (equal-molar Cl^−^, Br^−^, NO_3_^−^, and SO_4_^2−^), as well as trace iodine solutions (5 ppm and 100 ppb I_2_, I^−^, I_3_^−^) supplemented with a 10-fold excess of multiple co-existing competing anions (equal-molar Cl^−^, Br^−^, NO_3_^−^, and SO_4_^2−^), respectively was flowed through the column by a syringe pump at a flow rate of 0.3 mL min^−1^. The solution flowing through the sample-filled column was collected, and the remaining iodine concentration in the solution was measured using UV-Vis spectroscopy or ICP-MS.

**Trace level of iodine and iodide adsorption from aqueous solutions**

iPAF-TEPT was added to I_2_, I^−^, or I_3_^−^ aqueous solution with 5 ppm and 100 ppb, supplemented with a 10-fold excess of multiple co-existing competing anions (equal-molar Cl^−^, Br^−^, NO_3_^−^, and SO_4_^2−^) respectively, stirred at a rate of 1000 rpm at room temperature, sampled, and filtered using a 0.2 μm PES membrane at different times. The residual iodine concentration in the filtrate was measured by ICP-MS. Additionally, the trace iodine removal method conducted under acidic conditions (pH = 1−6) was the same as described above.

**Recyclable performance experiments**

The adsorbent was immersed in the aqueous solution (1.2 mM I_2_, 0.4 mM I^−^, 0.4 mM I_3_^−^, 5 ppm I_2_, 5 ppm I^−^, 5 ppm I_3_^−^, 100 ppb I_2_, 100 ppb I^−^, or 100 ppb I_3_^−^, 1 mg mL^−1^), stirred at a rate of 1000 rpm at room temperature, sampled at different times, and filtered using a 0.2 μm PES membrane until the concentration of iodine remaining in the filtrate remained unaltered. The residual iodine concentration in the filtrate was measured using UV-Vis spectroscopy or ICP-MS and filtered to obtain I_2_@iPAF-TEPT, I^−^@iPAF-TEPT, and I_3_^−^@iPAF-TEPT. Thereafter, I_2_@iPAF-TEPT, I^−^@iPAF-TEPT or I_3_^−^@iPAF-TEPT was added to a beaker filled with ethanol and ultrasonicated for 4 hours. The regenerated samples were filtered and vacuum-dried at 40 °C overnight. The recycled adsorbent was used for at least five subsequent cycles. Additionally, 10 equivalents of competing anions (Cl^−^, Br^−^, NO_3_^−^, SO_4_^2−^) were added to the 5 ppm and 100 ppb I_2_, I^−^, and I_3_^−^ aqueous solutions to further investigate the cyclic performance of iPAF-TEPT, with the experimental method being the same as described above.

**Computational method**

The binding energies (eV) between the PAF and the polyiodide species at the B3LYP level of exchange functional of the DFT calculations. All the structures were calculated using the defv2svp[3] basis set. The binding energy between A and B was calculated using ΔE = Etot − EA − EB, where EA and EB are the energy of A and B, respectively, while Etot is the energy of the adduct.

The theoretical calculations were performed using the Gaussian 16 suite of programs.^[3]^ Structures of the studied molecule and its complexes with I^−^ were fully optimized at the B3LYP-D3BJ/def2-SVP level of theory. The vibrational frequencies of the optimized structures were measured at the same level. The structures were characterized as a local energy minimum on the potential energy surface by verifying that all vibrational frequencies were real. The Gauss View package was used to plot color-filled iso-surface graphs to visualize the molecular electrostatic potential (MESP). ^[4]^

**
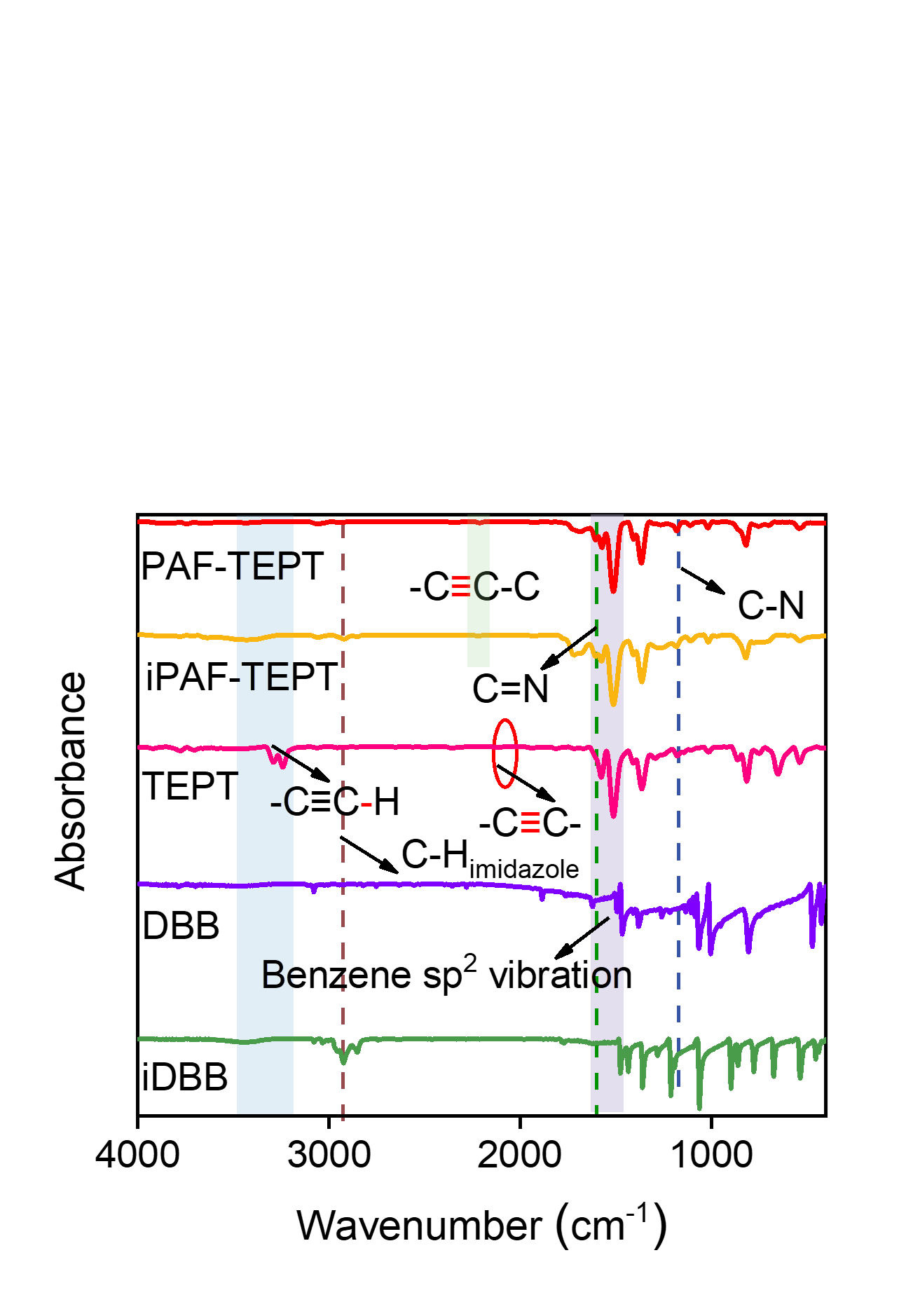
**

**Figure S3.** FT-IR spectra of iPAF-TEPT and PAF-TEPT.


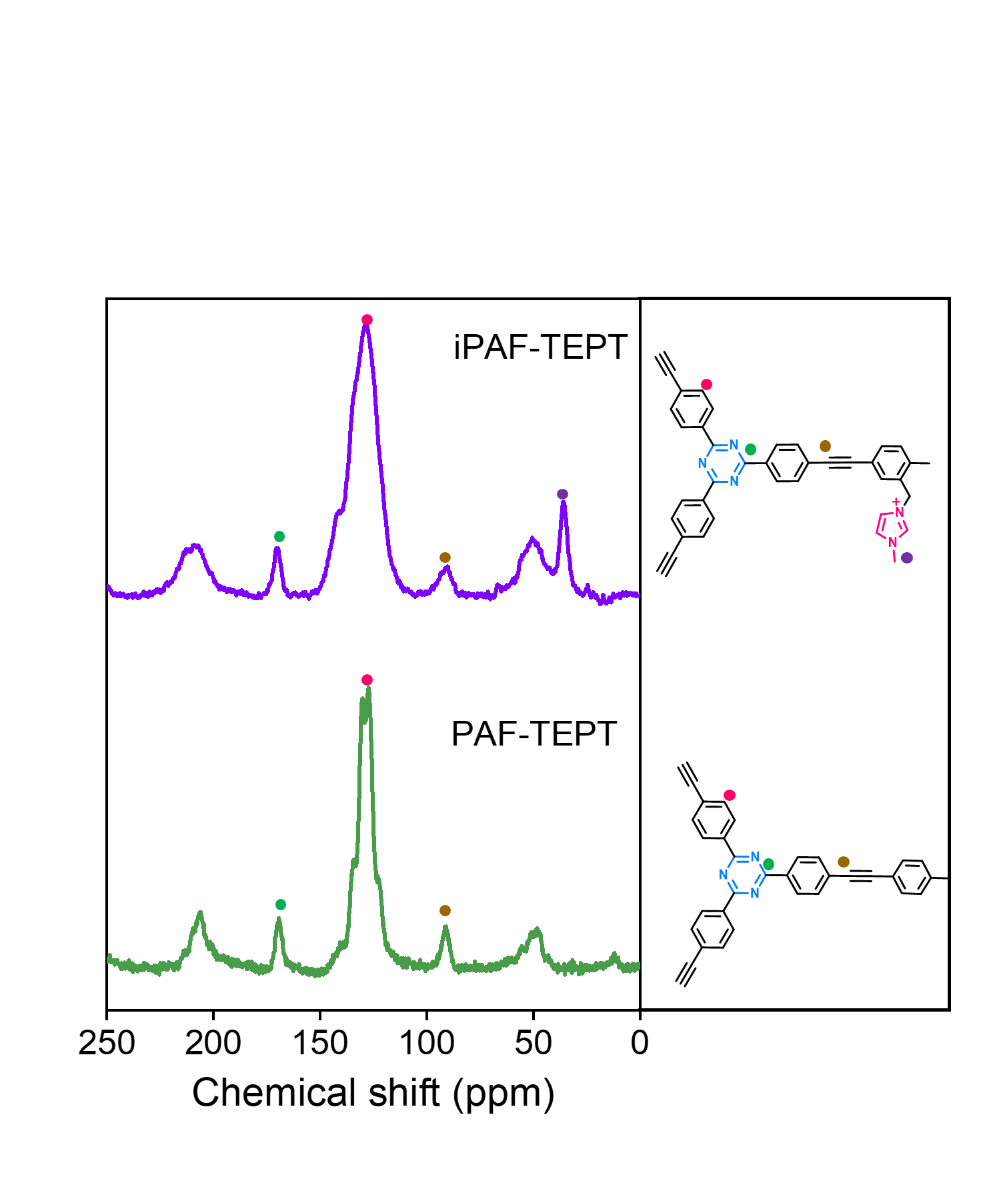


**Figure S4.** ^13^C NMR of iPAF-TEPT and PAF-TEPT.


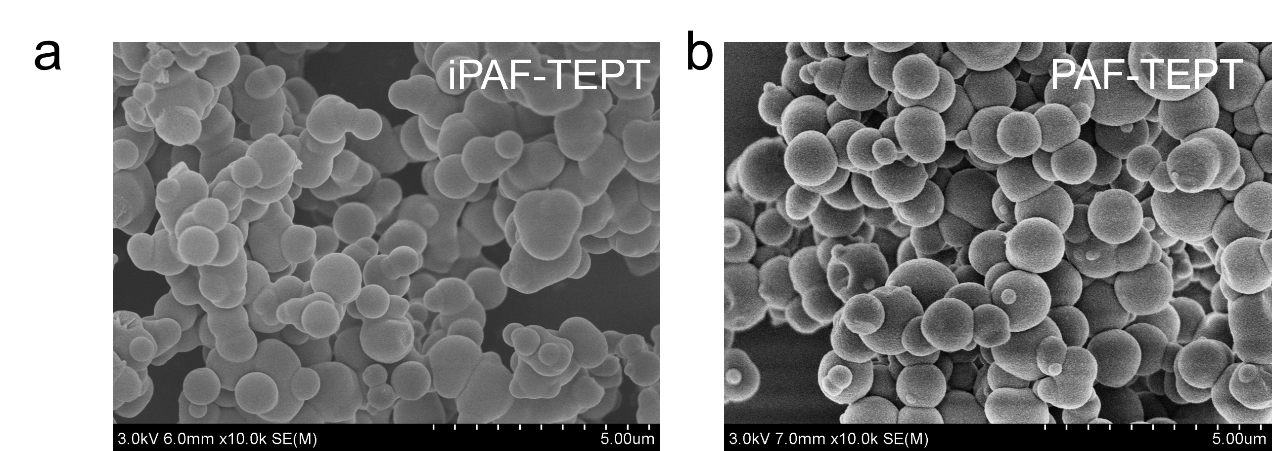


**Figure S5.** SEM images of two adsorbents. a) iPAF-TEPT. b) PAF-TEPT.


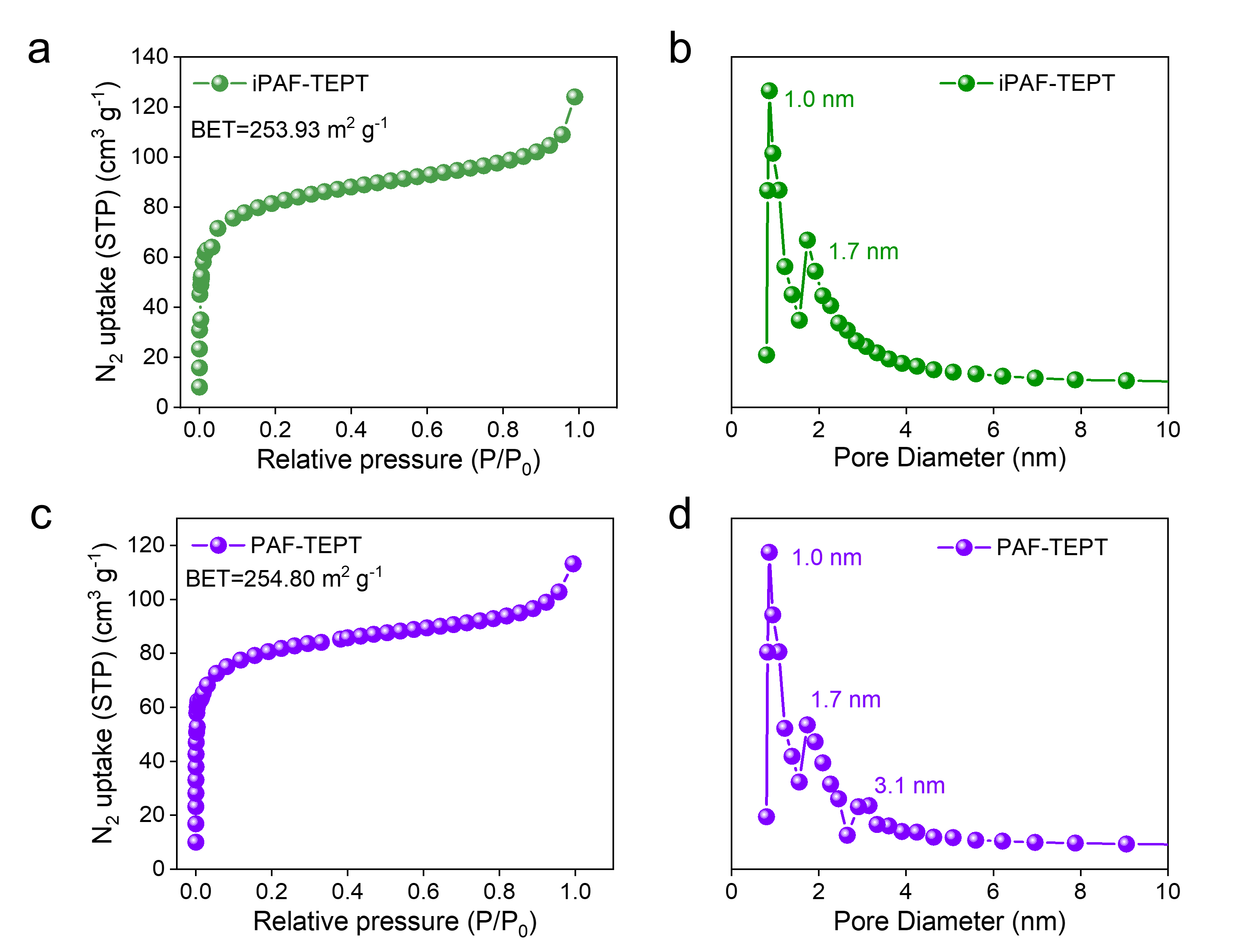


**Figure S6.** N_2_ adsorption and desorption curves and pore size distribution of two adsorbents. a) b) iPAF-TEPT. c) d) PAF-TEPT.


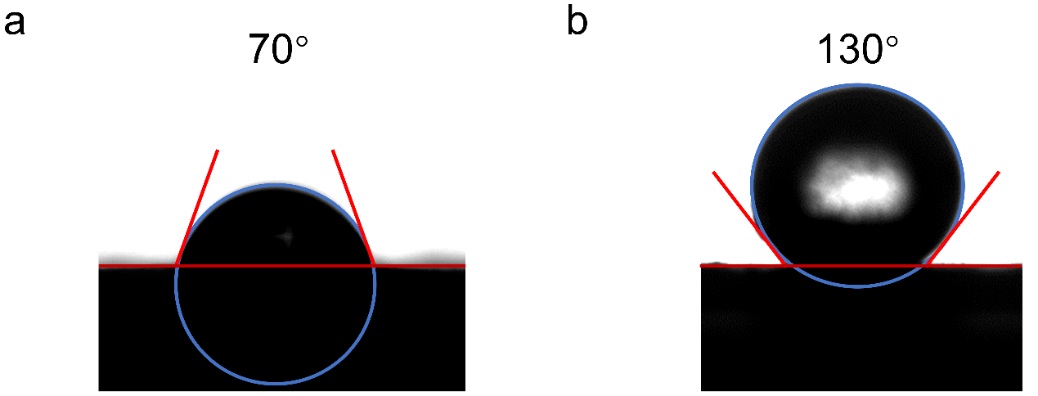


**Figure S7.** Water contact angle of iPAF-TEPT and PAF-TEPT. a) iPAF-TEPT. b) PAF-TEPT.


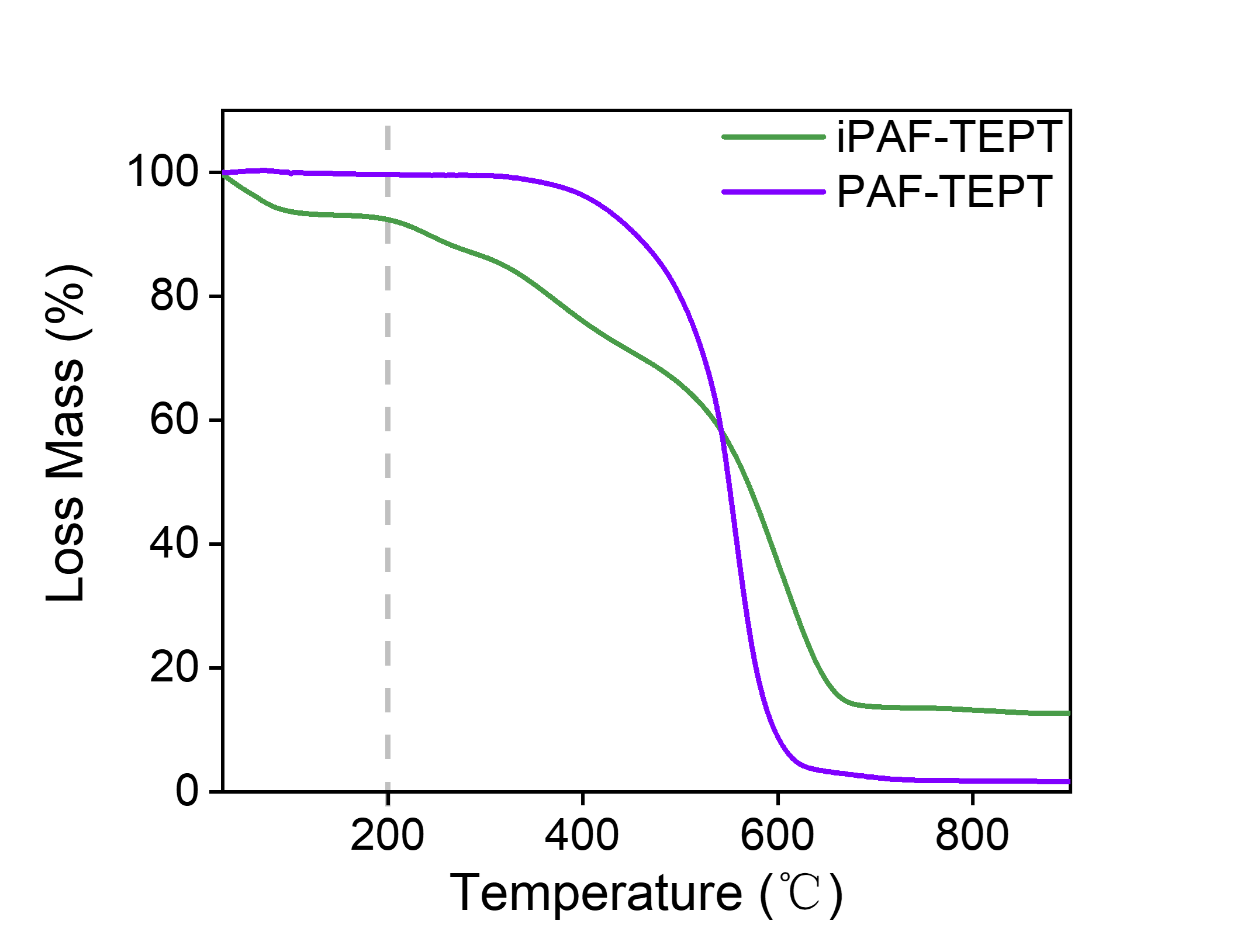


**Figure S8.** TGA curves of iPAF-TEPT and PAF-TEPT.


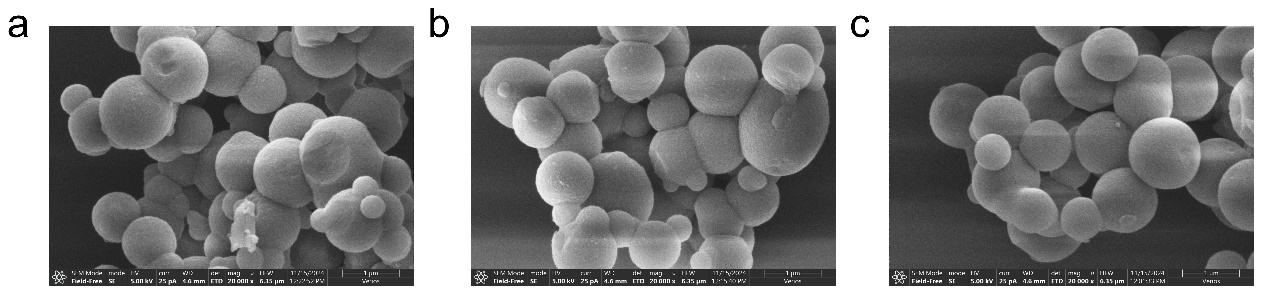


**Figure S9.** SEM images of iPAF-TEPT after being treated with acid, alkali, and water immersion. a) After being soaked in 6 M HCl for 48 h. b) After being soaked in 6 M NaOH for 48 h. c) After being soaked in water for 30 d.


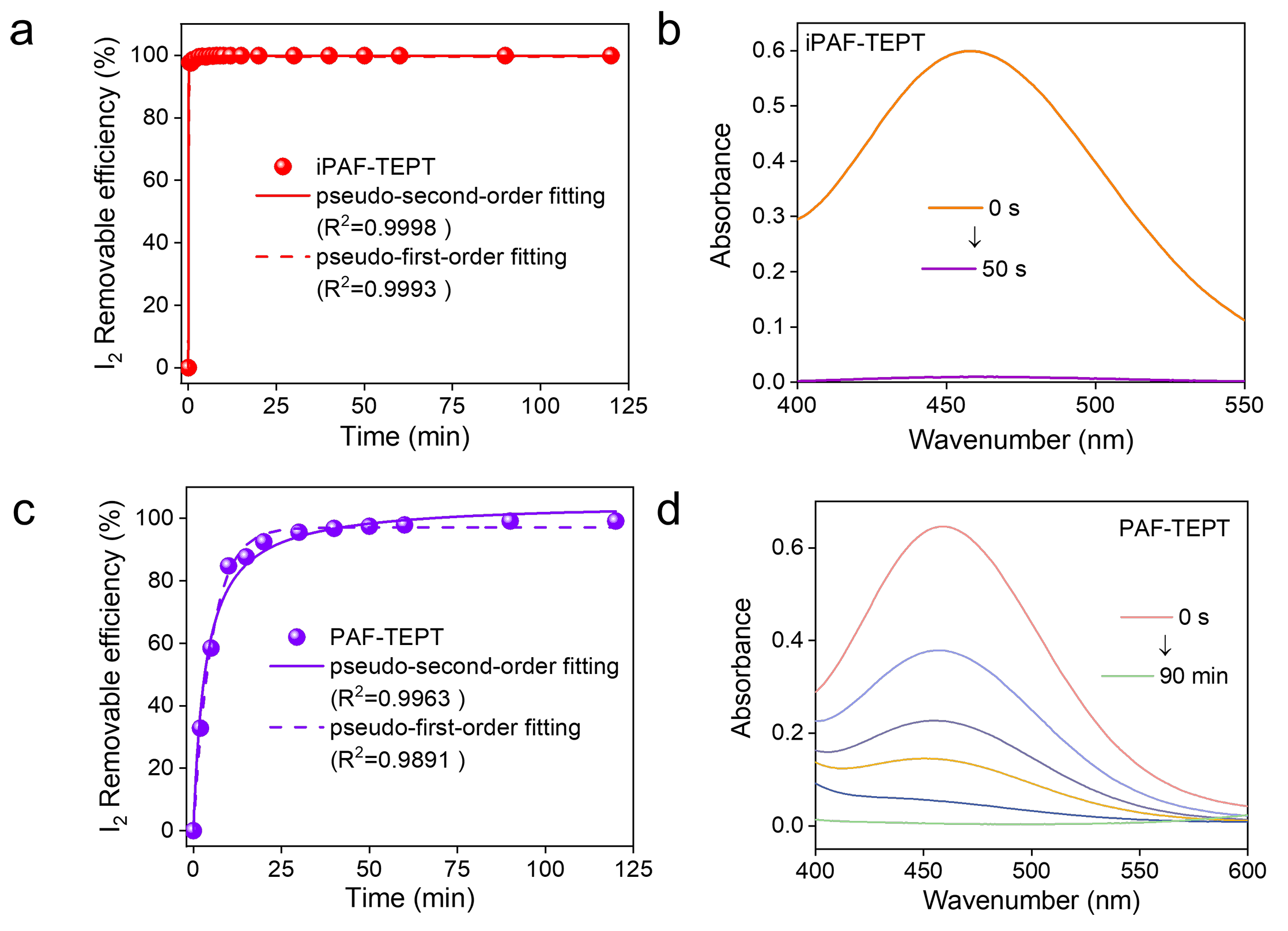


**Figure S10.** Pseudo-first and pseudo-second order kinetics and UV-Vis spectra of 1.2 mM I_2_ aqueous solution of two adsorbents. a) b) iPAF-TEPT. c) d) PAF-TEPT.


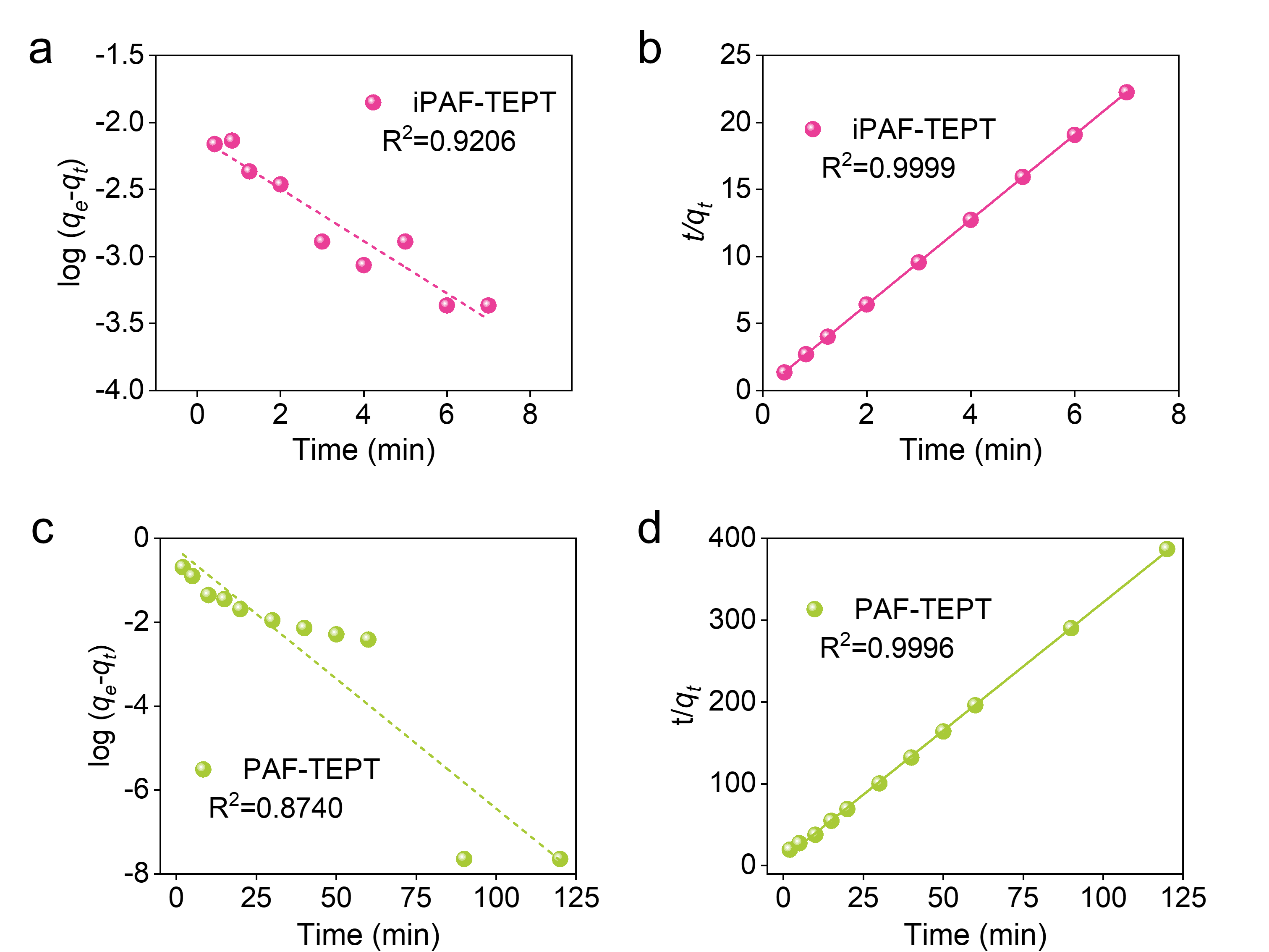


**Figure S11.** Linear fitting of the adsorption kinetic of iPAF-TEPT and PAF-TEPT to iodine in 1.2 mM I_2_ aqueous solution. a) Pseudo-first-order kinetic linear fitting of iPAF-TEPT. b) Pseudo-second-order kinetic linear fitting of iPAF-TEPT. c) Pseudo-first-order kinetic linear fitting of PAF-TEPT. d) Pseudo-second-order kinetic linear fitting of PAF-TEPT.


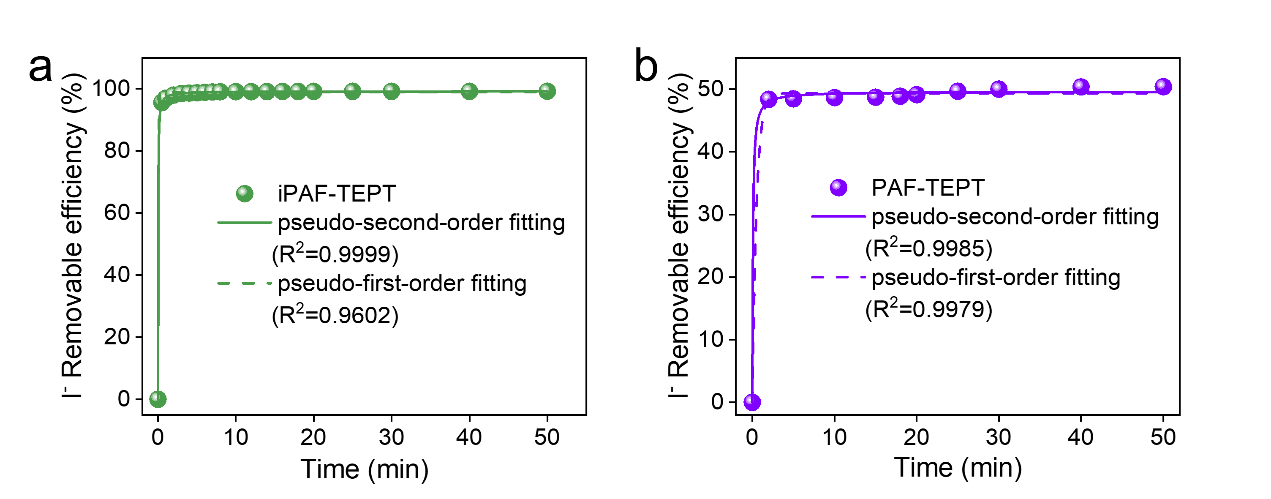


**Figure S12.** Pseudo-first and pseudo-second order kinetics of 0.4 mM I^−^ aqueous solution of two adsorbents. a) iPAF-TEPT. b) PAF-TEPT.


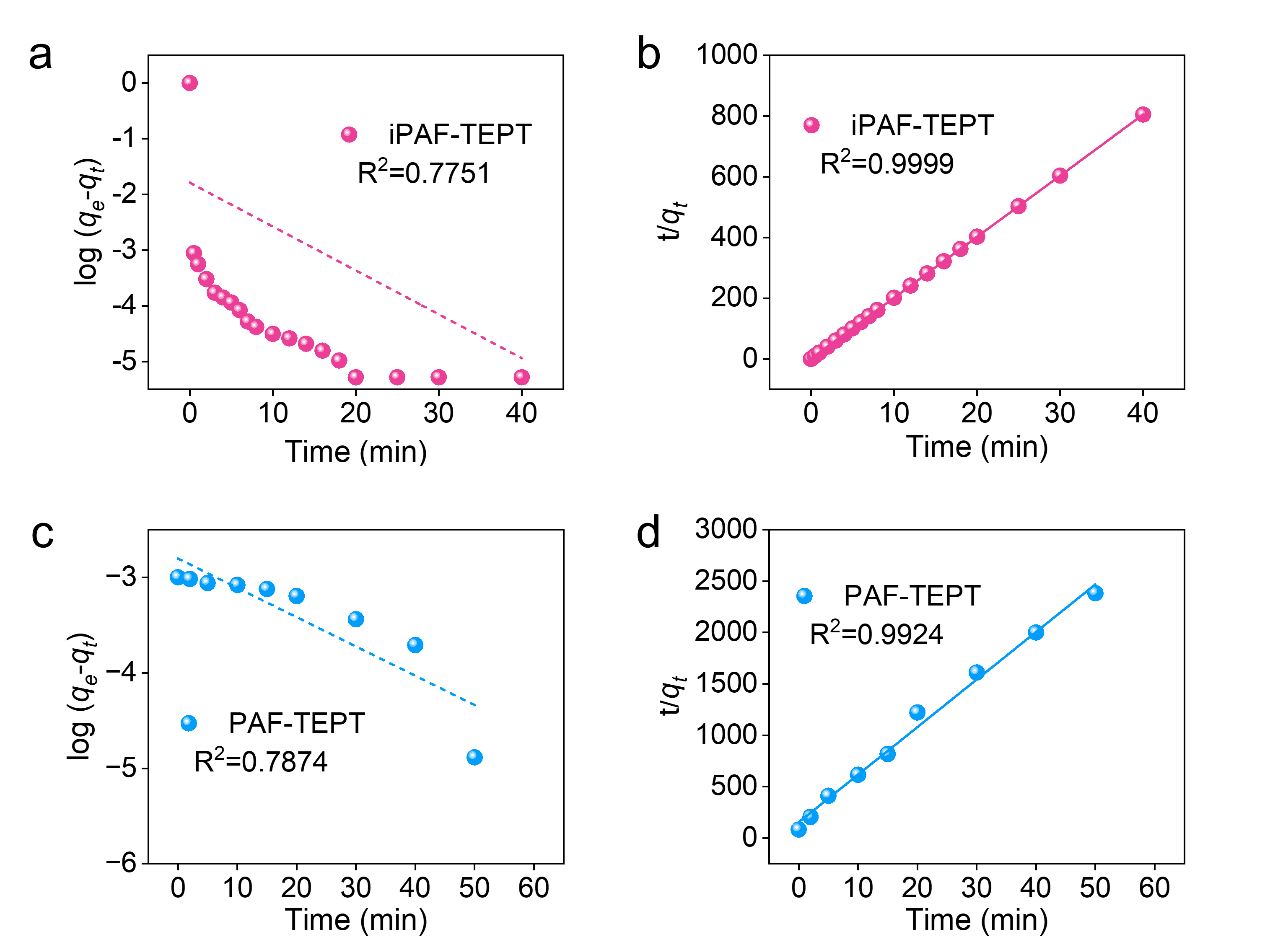


**Figure S13.** Linear fitting of the adsorption kinetic of iPAF-TEPT and PAF-TEPT to iodine in 0.4 mM I^−^ aqueous solution. a) Pseudo-first-order kinetic linear fitting of iPAF-TEPT. b) Pseudo-second-order kinetic linear fitting of iPAF-TEPT. c) Pseudo-first-order kinetic linear fitting of PAF-TEPT. d) Pseudo-second-order kinetic linear fitting of PAF-TEPT.


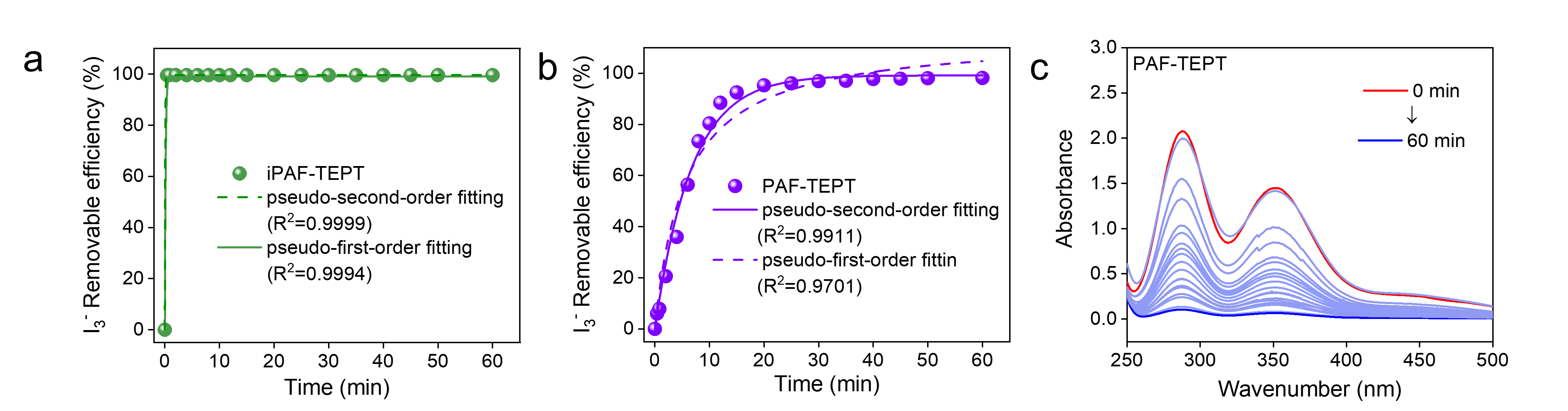


**Figure S14.** Pseudo-first and pseudo-second order kinetics and UV-Vis spectra of 0.4 mM I_3_^−^ aqueous solution of two adsorbents. a) iPAF-TEPT. b) c) PAF-TEPT.


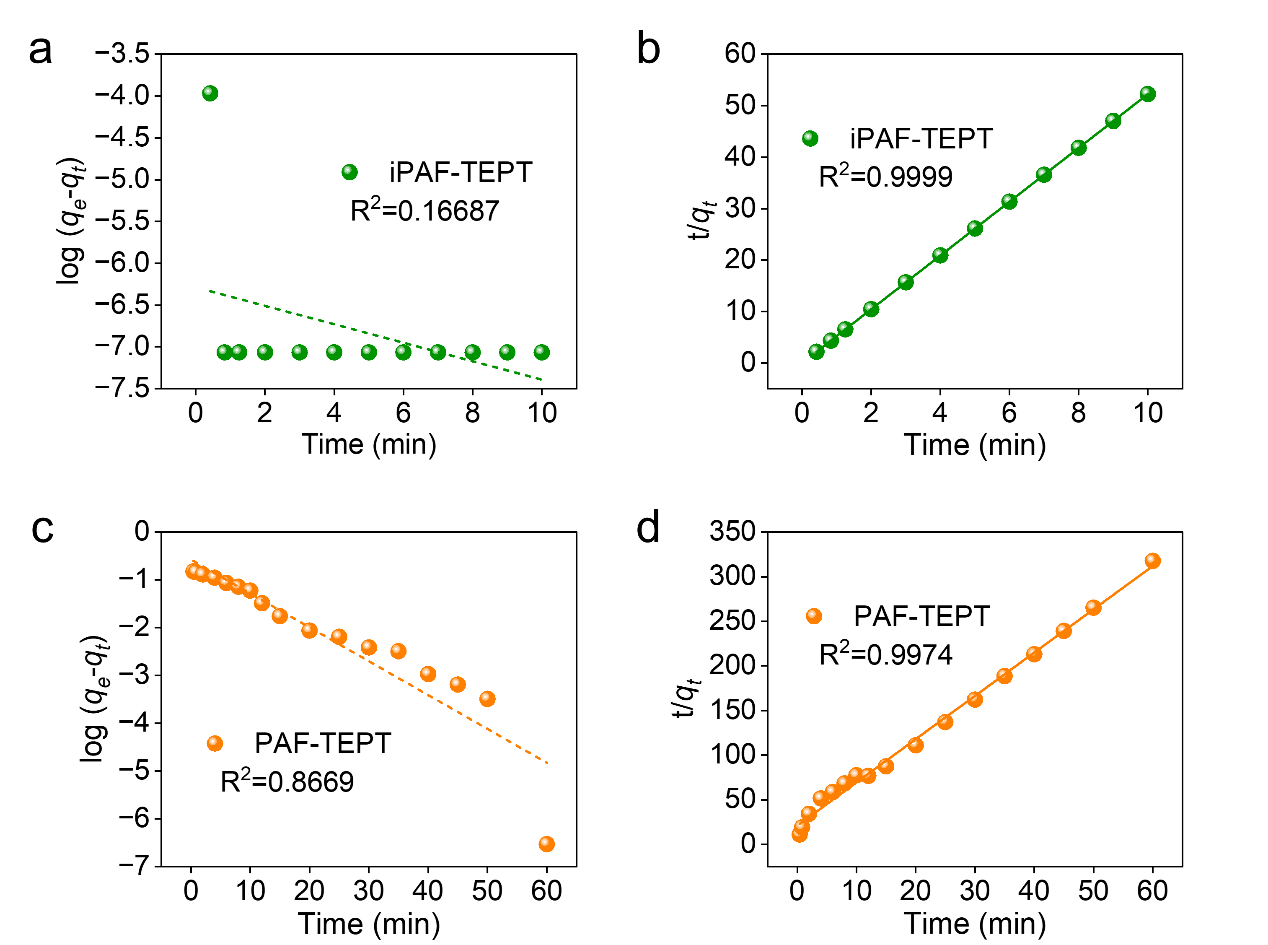


**Figure S15.** Linear fitting of the adsorption kinetic of iPAF-TEPT and PAF-TEPT to iodine in 0.4 mM I_3_^−^ aqueous solution. a) Pseudo-first-order kinetic linear fitting of iPAF-TEPT. b) Pseudo-second-order kinetic linear fitting of iPAF-TEPT. c) Pseudo-first-order kinetic linear fitting of PAF-TEPT. d) Pseudo-second-order kinetic linear fitting of PAF-TEPT.


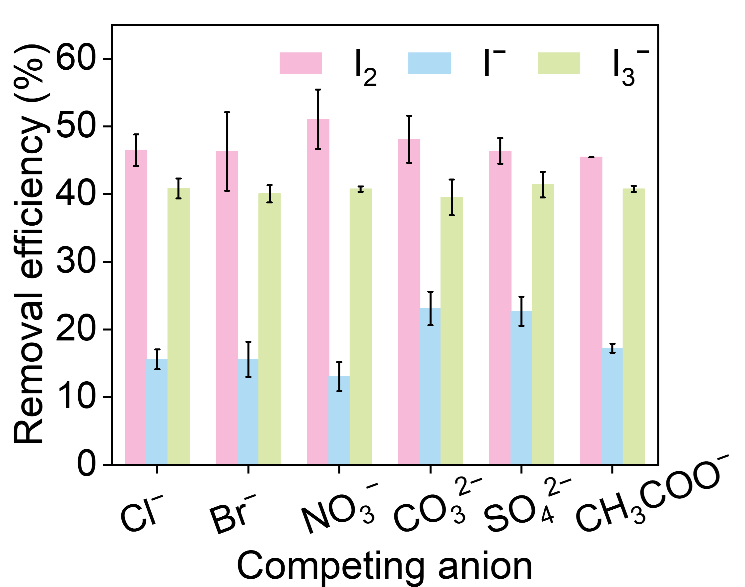


**Figure S16.** Removal efficiency of PAF-TEPT at 1000 equivalent competing anion.


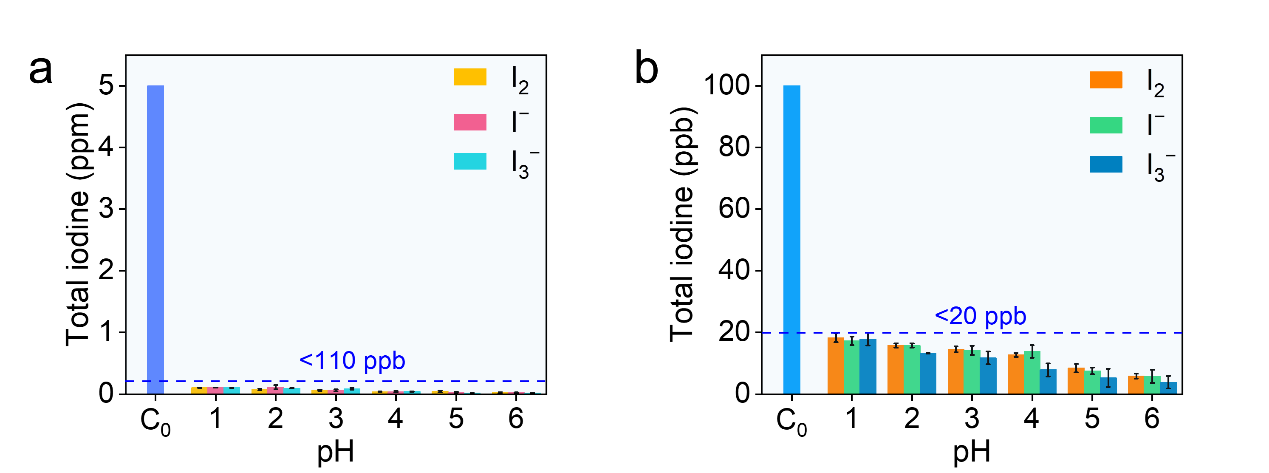


**Figure S17.** Adsorption of different iodine species from aqueous solutions and aqueous solutions containing a mixture of 10 equivalent anions, with initial iodine species concentrations of 5 ppm and 100 ppb. a) 5 ppm I_2_, I^−^, and I_3_^−^ solution. b) 100 ppb I_2_, I^−^, and I_3_^−^ solution.


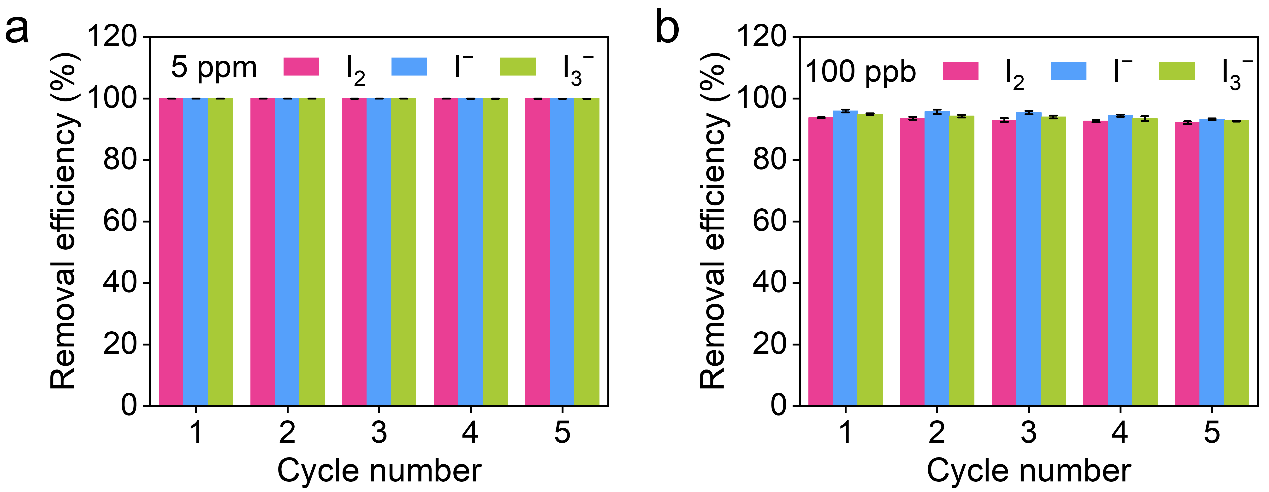


**Figure S18.** Reusability for different trace iodine species removal in aqueous solution. a) 5 ppm I_2_, I^−^, or I_3_^−^ solution. b) 100 ppb I_2_, I^−^, or I_3_^−^ solution.


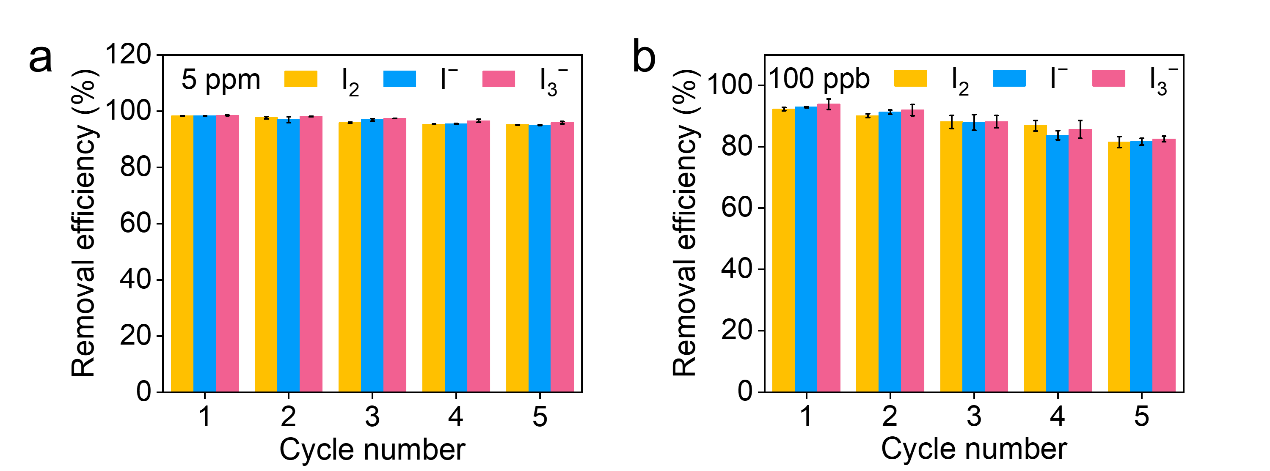


**Figure S19.** Reusability of the adsorbent for different trace iodine species in the presence of 10 equivalent concentrations of competing anions (Cl^−^, Br^−^, NO_3_^−^, SO_4_^2−^) in aqueous solution. a) 5 ppm I_2_, I^−^, and I_3_^−^ solution. b) 100 ppb I_2_, I^−^, and I_3_^−^ solution.


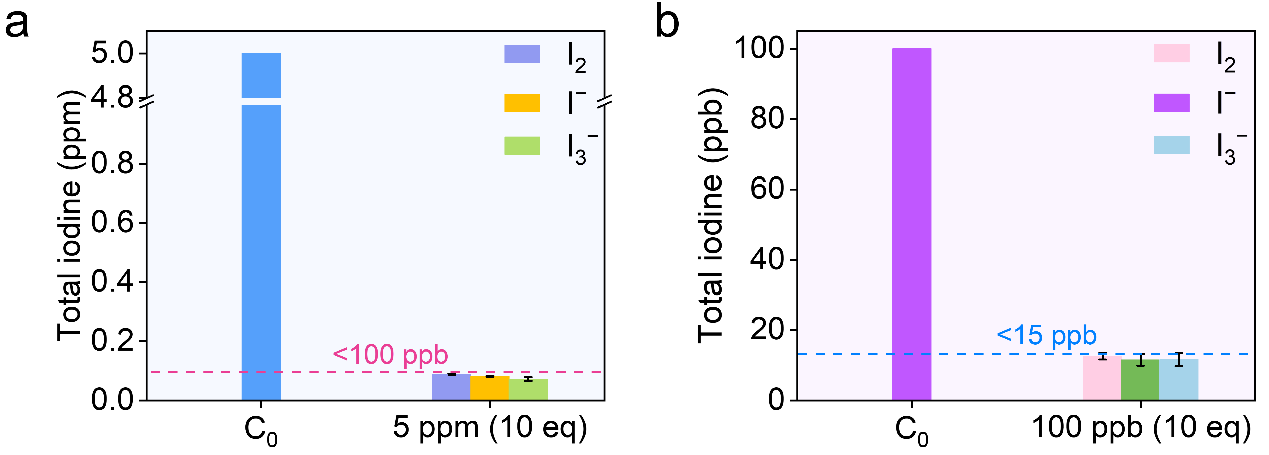


**Figure S20.** Column breakthrough adsorption ability to different trace iodine species in the presence of 10 equivalent co-existing competing anions (Cl^−^, Br^−^, NO_3_^−^, SO_4_^2−^). a) 5 ppm I_2_, I^−^, and I_3_^−^ solution. b) 100 ppb I_2_, I^−^, and I_3_^−^ solution.


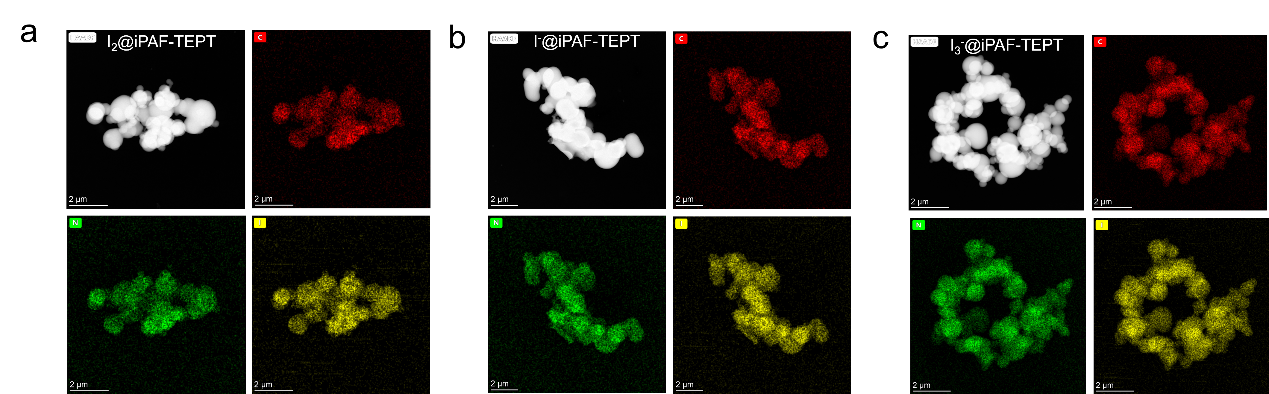


**Figure S21.** TEM-EDS of iodine-loaded iPAF-TEPT. a) I_2_@iPAF-TEPT. b) I^−^@iPAF-TEPT. c) I_3_^−^@iPAF-TEPT.


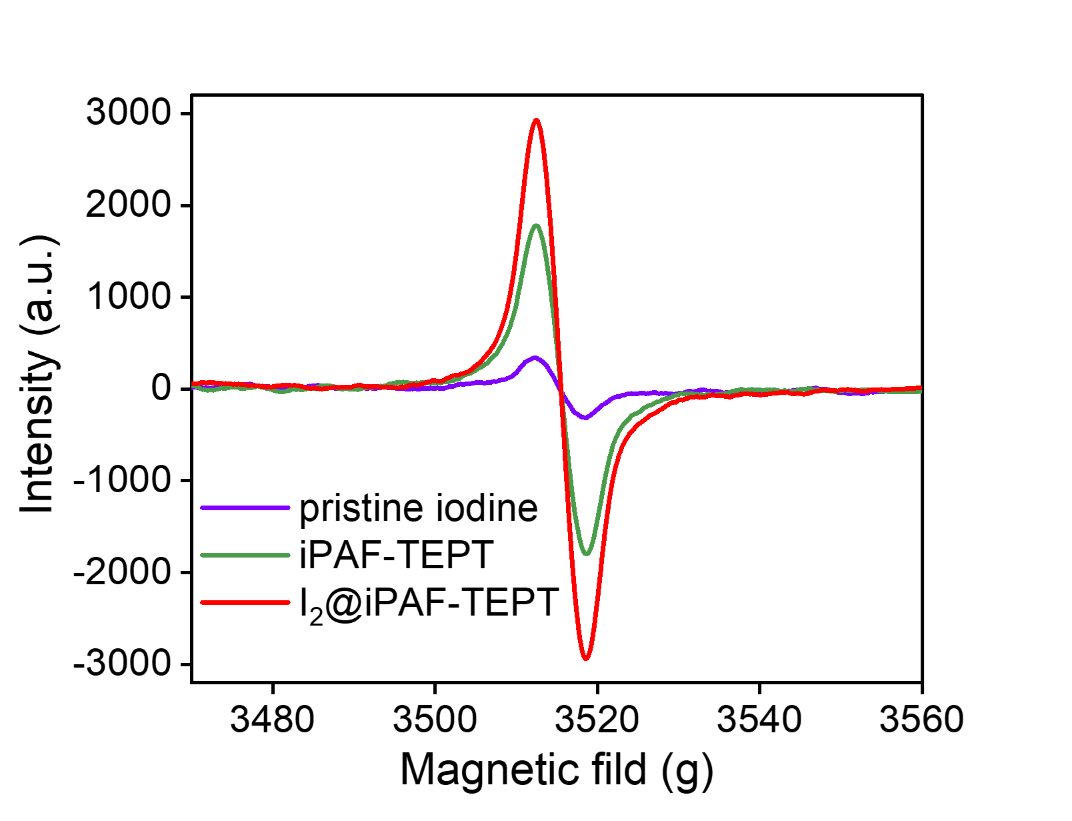


**Figure S22.** EPR of pristine iodine, iPAF-TEPT, I_2_@ iPAF-TEPT.

**Table S1.** Experiments on the adsorption capacity of different adsorbents for iodine involve I_2_ aqueous solutions.

| Type | Materials | Iodine uptake capacity (g g^−1^) | Reference |
| --- | --- | --- | --- |
| PAF | iPAF-TEPT | 15.59 | **This work** |
| NAS | SUPE–py–Imine–Cage | 8.41 | [5] |
| NAS | SUPE–py–Amine–Cage | 7.06 | [5] |
| COF | COFA–1 | 6.78 | [6] |
| COF | COFP–1 | 5.05 | [6] |
| POP | C[4]P-HEPM | 4.45 | [7] |
| POP | C[4]P-TEPM | 4.02 | [7] |
| POP | C[4]P-BTP | 3.03 | [8] |
| POP | C[4]P-TPE | 2.80 | [8] |
| MOF | MOF nanosheets | 2.32 | [9] |
| POP | C[4]P-BP | 2.31 | [8] |
| POP | C[4]P-TTP | 2.22 | [8] |
| NM | Mg–Co–Al–LDH@g– C3N4 | 2.20 | [10] |
| POP | C[4]P-BT | 2.16 | [8] |
| NM | Fe_3_O_4_@PPy | 1.63 | [11] |
| Carbon | PTEP | 1.45 | [12] |
| POP | C[4]P-DPP | 1.35 | [8] |
| MOF | N-MOF-PAN fibers | 1.34 | [13] |
| PAF | PAF-TEPT | 1.22 | **This work** |
| POP | C-poly-1_20_ | 1.09* | [14] |
| COF | TAPE-BPDA COF | 0.99 | [15] |
| POP | C-poly-1_50_ | 0.92* | [14] |
| calixpyridinium | PTIBBL | 0.67 | [16] |
| POP | C-poly-1_80_ | 0.66* | [14] |
| POP | C-poly-1_100_ | 0.50* | [14] |
| POP | calixpyridinium-PyTS | 0.445 | [17] |
| POP | Fc-PP-POP | 0.10 | [18] |
| trianglamine(Δ) powder | Δ@PPG3 | 0.04 | [19] |
| trianglamine(Δ) powder | Δ@PPG6 | 0.04 | [19] |

Note: COF: covalent-organic framework; POP: porous organic polymers; MOF: metal organic framework; PAF: Porous aromatic framework. NAS: nonporous amorphous sorbents; NM: Nanoscale materials; * Calculated according to literature data.

**Table S2.** Experiments on the adsorption capacity of different adsorbents for iodine involve I_3_^−^ aqueous solutions.

| Type | Materials | Iodine uptake capacity (g g^−1^) | Reference |
| --- | --- | --- | --- |
| PAF | iPAF-TEPT | 14.68 | **This work** |
| NAS | SUPE-py-Imine-Cage | 9.01 | [5] |
| NAS | SUPE-py-Amine-Cage | 8.10 | [5] |
| COF | COFA-1 | 7.13 | [6] |
| POP | PP-3 | 5.90 | [20] |
| POP | PP-2 | 5.80 | [20] |
| POP | PP-4 | 5.10 | [20] |
| COF | COFP-1 | 4.81 | [6] |
| POP | C[4]P-HEPM | 4.45 | [7] |
| POP | PP-1 | 4.20 | [20] |
| POP | C[4]P-TEPM | 4.02 | [7] |
| POP | PP-5 | 4.00 | [20] |
| MOF | MOF nanosheets | 3.70 | [9] |
| HOF | HcOF-4 | 3.57 | [21] |
| MOF | N-MOF-PAN fibers | 3.56 | [13] |
| POP | Compound 1 | 3.55 | [22] |
| POP | C[4]P-BTP | 3.24 | [8] |
| HOF | HcOF-2 | 3.23 | [21] |
| POP | CaCOP3 | 3.10 | [23] |
| POP | Compound 2 | 3.04 | [22] |
| HOF | HcOF-3 | 3.00 | [21] |
| POP | C[4]P-TPE | 2.99 | [8] |
| POP | CaCOP2 | 2.81 | [23] |
| POP | C[4]P-TTP | 2.51 | [8] |
| POP | CaCOP1 | 2.40 | [23] |
| POP | C[4]P-BP | 2.37 | [8] |
| POP | C[4]P-BT | 2.32 | [8] |
| POP | CalCOP1 | 2.32 | [24] |
| HOF | HcOF-1 | 2.10 | [25] |
| POP | CalCOP2 | 1.76 | [24] |
| POP | C[4]P-DPP | 1.58 | [8] |
| MOF | {[Mn_2_(oxdz)_2_(tpbn)(H_2_O)_2_]·2C_2_H_5_OH}_n_ | 1.10 | [26] |
| aluminum macrocycle-faced cages | AIMC-1 | 0.89 | [27] |
| PAF | PAF-TEPT | 0.83 | **This work** |
| POP | CalCOP3 | 0.35 | [24] |
| MOF | SCNU-Z4 | 0.33 | [28] |
| POP | CalCOP4 | 0.15 | [24] |

Note: HOF: Hydrogen-bonded cross-linked organic frameworks.

**Reference**

[1] Y. Ma, F. C. Cui, H. Z. Rong, J. Song, X. F. Jing, Y. Y. Tian, G. S. Zhu, *Angew. Chem. Int. Ed.* **2022**, 61, e202113682.

[2] C. I. Pearce, E. A. Cordova, W. L. Garcia, S. A. Saslow, K. J. Cantrell, J. W. Morad, O. Qafoku, J. Matyas, A. E. Plymale, S. Chatterjee, J. Kang, F. C. Colon, T. G. Levitskaia, M. J. Rigali, J. E. Szecsody, S. M. Heald, M. Balasubramanian, S. Wang, D. T. Sun, W. L. Queen, R. Bontchev, R. C. Moore, V. L. Freedman, *Sci. Total Environ.* **2020**, 716, 136167.

Queen, R. Bontchev, R. C. Moore, V. L. Freedman, Sci. Total Environ. 2020, 716, 136167.

[3] M. Frisch, G. Trucks, H. B. Schlegel, G. E. Scuseria, M. Robb, J. R. Cheeseman, G. Scalmani, V. Barone, G. A. Petersson, H. Nakatsuji, X. Li, M. Caricato, A. Marenich, J. Bloino, Benjamin G. Janesko, R. Gomperts, B. MENNUCCI, H. Hratchian, J. V. Ortiz, A. Izmaylov, J. Sonnenberg, D. Williams-Young, F. Ding, F. Lipparini, F. Egidi, J. Goings, B. Peng, A. Petrone, T. Henderson, D. Ranasinghe, Viatcheslav G. Zakrzewski, J. GAO, N. Rega, G. Zheng, W. Liang, M. Hada, M. Ehara, K. Toyota, R. Fukuda, J. Hasegawa, M. Ishida, T. Nakajima, Y. Honda, O. Kitao, H. Nakai, T. Vreven, K. Throssell, J. Montgomery, J. Peralta, F. Ogliaro, M. Bearpark, J. J. Heyd, E. Brothers, K. Kudin, V. Staroverov, T. Keith, R. Kobayashi, J. Normand, K. Raghavachari, A. P. Rendell, J. C. Burant, S. Iyengar, J. Tomasi, M. Cossi, J. M. Millam, M. Klene, C. Adamo, R. Cammi, J. W. Ochterski, R. A. Martin, K Morokuma, O. Farkas, J. B. Foresman, D. Fox, H. B. Schlegel, G. Scalmani, G. A. Petersson, B. Mennucci, H. Hratchian, J. V. Ortiz, A. Izmaylov, F Lipparini, J Goings, B Peng, Vyacheslav G. Zakrzewski, J. Gao, G Zheng, W. Liang, R. Fukuda, J. Hasegawa, M. Ishida, Y. Honda, O. Kitao, H. Nakai, T. Vreven, K. Throssell, J. A. Montgomery Jr, F Ogliaro, M. Bearpark, V. Staroverov, R. Kobayashi, M. Cossi, C. Adamo, R. D. Martin, J. Foresman, M. Frisch, G. Trucks, H. Schlegel, G. Scuseria, M. Robb, J. Cheeseman, G. Petarsson, X. Li, A. Marenich, B. Janesko, H. Hratchian, J. Ortiz, A. Izmaylov, J. Sonnenberg, F. Ding, T. Henderson, V. Zakrzewski, M Hada, J. Montgomery, J. Peralta, M. Bearpark, J. Heyd, E. Brothers, K. Kudin, V. Staroverov, T. Keith, K. Raghavachari, A. Rendell, J. Burant, S. Iyengar, J. Tomasi, J. Millam, M. Klene, R. Cammi, J. Ochterski, R. Martin, O. Farkas, J. Foresman, D. Fox, M. Frisch, G. Trucks, V. Barone, J. Bloino, R. Gomperts, A. Petrone, T. Henderson, W. Liang, M. Ishida, T. Nakajima, Y. Honda, H. Nakai, R. Kobayashi, J. C. Burant, S. Iyengar, J. Tomasi, D. Fox, A. Izmaylov, M. Ehara, J. A. Montgomery Jr., J. Millam, J. Knox, J. N. Cross, V. Bakken, J. Jaramillo, R. E. Stratmann, O. Yazyev, A. Austin, C. Pomelli, G. Voth, P. Salvador, J. J. Dannenberg, S Dapprich, A. Daniels, J. Cioslowski, J. Gao, J. J. Heyd, J. Foresman, N. Rega, G. Zheng, K. Toyota, T. Nakajima, J. Montgomery, E. Brothers, K. Raghavachari, R. Martin, J. Frisch M., J. Peralta, K. A. Kudin, Alistair Rendell, J. Cross, V. Bakken, J. Jaramillo, V. Barone, Alistair Rendell, J. V. Oiz, Williams, J. A. Montgomery, J. Zheng, E. Brothers, K. N. Kudin, J. C. T. Rendell, S. Burant, A. Austin, P. Salvador, M.J. Frisch, W. Li, J. Montgomery, Jr., M. J. Frisch, G. W. Trucks, H. B. Schlegel, G. E. Scuseria, M. A. Robb, J. R. Cheeseman, G. A. Petersson, A. V. Marenich, B. G. Janesko, H. P. Hratchian, J. V. Ortiz, A. F. Izmaylov, J. L. Sonnenberg, V. G. Zakrzewski, J. J. A. Montgomery, J. E. Peralta, M. J. Bearpark, J. J. Heyd, E. N. Brothers, K. N. Kudin, V. N. Staroverov, T. A. Keith, A. P. Rendell, J. C. Burant, S. S. Iyengar, J. M. Millam, J. W. Ochterski, R. L. Martin, J. B. Foresman, D. J. Fox., Gaussian 16 Revision. A.03, Gaussian Inc., Wallingford, CT, **2016**.

[4] R. I. Dennington, T. Keith, J. Millam, GaussView, Version 6.0.16, Semichem. Inc, Shawnee Mission, KS, **2008**.

[5] W. Zhou, A. Li, M. Zhou, Y. Xu, Y. Zhang, Q. He, *Nat. Commun.* **2023**, 14, 5388.

[6] X. F. Li, Z. M. Jia, J. Zhang, Y. D. Zou, B. Jiang, Y. D. Zhang, K. W. Shu, N. Liu, Y. Li, L. J. Ma, *Chem. Mat.* **2022**, 34, 11062.

[7] Z. Y. Zheng, Q. Y. Lin, L. H. Xie, X. L. Chen, H. Zhou, K. H. Lin, D. S. Zhang, X. D. Chi, J. L. Sessler, H. Y. Wang, *J. Mater. Chem. A.* **2023**, 11, 13399.

[8] L. Xie, Z. Zheng, Q. Lin, H. Zhou, X. Ji, J. L. Sessler, H. Wang, *Angew. Chem. Int. Ed.* **2022**, 61, e202113724.

[9] C. X. Yu, X. J. Li, J. S. Zong, D. J. You, A. P. Liang, Y. L. Zhou, X. Q. Li, L. L. Liu, *Inorg. Chem.* **2022**, 61, 13883.

[10] E. Yazdankish, M. Foroughi, M. H. A. Azqhandi, *J. Hazard. Mater.* **2020**, 389, 122151.

[11] D. K. L. Harijan, V. Chandra, T. Yoon, K. S. Kim, *J. Hazard. Mater.* **2018**, 344, 576.

[12] Y. C. Yin, Y. Yang, G. J. Liu, H. L. Chen, D. Gong, Y. M. Ying, J. R. Fan, S. J. Liu, Z. Li, C. H. Wang, Z. Y. Guo, Z. K. Li, C. B. Yu, G. F. Zeng, Chem. Eng. J. 2022, 441, 135996.

[13] D. Y. Chen, T. T. Ma, X. Y. Zhao, X. F. Jing, R. Zhao, G. S. Zhu, *Acs Appl Mater Inter* **2022**, 14, 47126.

[14] X. H. Xu, Y. X. Li, L. Zhou, N. Liu, Z. Q. Wu, *Chem. Sci.* **2022**, 13, 1111.

[15] R. Chen, T. L. Hu, Y. Q. Li, *React Funct Polym* **2021**, 159, 104806.

[16] M. Huang, L. Yang, X. Y. Li, G. J. Chang, *Chem. Commun.* **2020**, 56, 1401.

[17] K. Wang, G. J. Gao, X. Y. Wang, M. M. Wang, H. X. Dou, *Langmuir* **2021**, 37, 11422.

[18] C. Miao, L. Chu, D. Guo, X. Ding, W. Guo, S. Wang, J. Sheng, J. Zhang, Z. Wang, B. Zhou, *J. Environ. Chem. Eng.* **2023**, 11, 110514.

[19] B. T. Benkhaled, A. Chaix, C. Gomri, S. Buys, N. Namar, N. Sehoulia, R. Jadhav, J. Richard, L. Lichon, C. Nguyen, M. Gary-Bobo, M. Semsarilar, *Acs Appl Mater Inter* **2023**, 12, 1900249.

[20] M. Avais, S. Chattopadhyay, *J Mater Chem A* **2022**, 10, 20090.

[21] X. F. Jiang, X. Z. Cui, A. J. E. Duncan, L. Li, R. P. Hughes, R. J. Staples, E. V. Alexandrov, D. M. Proserpio, Y. Y. Wu, C. F. Ke, *J. Am. Chem. Soc.* **2019**, 141, 10915.

[22] A. Sen, S. Sharma, S. Dutta, M. M. Shirolkar, G. K. Dam, S. Let, S. K. Ghosh, *Acs Appl Mater Inter* **2021**, 13, 34188.

[23] D. An, L. Li, Z. Z. Zhang, A. M. Asiri, K. A. Alamry, X. H. Zhang, *Mater Chem Phys* **2020**, 239, 122328.

[24] Z. Z. Zhang, L. Li, D. An, H. X. Li, X. H. Zhang, *J Mater Sci* **2020**, 55, 1854.

[25] Y. X. Lin, X. F. Jiang, S. T. Kim, S. B. Alahakoon, X. S. Hou, Z. Y. Zhang, C. M. Thompson, R. A. Smaldone, C. F. Ke, *J. Am. Chem. Soc.* **2017**, 139, 7172.

[26] A. Gogia, P. Das, S. K. Mandal, *Acs Appl Mater Inter* **2020**, 12, 46107.

[27] Y. J. Liu, Y. F. Sun, S. H. Shen, S. T. Wang, Z. H. Liu, W. H. Fang, D. S. Wright, J. Zhang, *Nat. Commun.* **2022**, 13, 10, 6632.

[28] G. Q. Wang, J. F. Huang, X. F. Huang, S. Q. Deng, S. R. Zheng, S. L. Cai, J. Fan, W. G. Zhang, *Inorg. Chem. Front.* **2021**, 8, 1083.
